# Soluble Fiber Intake Restores the Estrous Cycle and Sex Hormone Alterations in APP/PS1 Female Mice

**DOI:** 10.1007/s10571-026-01745-3

**Published:** 2026-05-20

**Authors:** Ivonne Sagrario Romero-Flores, Daniel Cuervo-Zanatta, Roberto Chavira, Vicente Sánchez-Valle, Jaime García-Mena, Claudia Perez-Cruz

**Affiliations:** 1 Pharmacology Department, Laboratory of Neuroplasticity and Neurodegeneration, Center for Research and Advanced Studies of the National Polytechnic Institute (Cinvestav), Av. IPN 2508, Mexico City, 07360 Mexico; 2 Genetics and Molecular Biology Department, Laboratory of reference and support for genomes, transcriptomes and microbiomes characterization, Center for Research and Advanced Studies of the National Polytechnic Institute (Cinvestav), Av. IPN 2508, Mexico City, 07360 Mexico; 3 Physiology of Reproduction, National Autonomous University of Mexico (UNAM), National Institute of Medical Science and Nutrition “Salvador Zubiran” (INCMNSZ), Mexico City, 14080 Mexico

## Abstract

Alzheimer’s disease (AD) is the most common form of dementia, with a higher prevalence in women than in men. It has been suggested that the decline in estrogen production after menopause may increase the risk of developing dementia. We have previously shown that transgenic female APP/PS1 mice, overexpressing APP and PS1 proteins, develop spontaneous gut dysbiosis with a consequent dysfunctional estrobolome leading to higher estradiol excretion rate compared to age-matched wildtype (WT) females. It has been demonstrated that soluble fiber intake restores gut microbiota dysbiosis, reduces anxiety and improves cognitive function in APP/PS1 male mice. Therefore, the aim of this study was to evaluate if the intake of soluble fiber can modify the gut microbiota composition, alleviating the estrobolome dysfunction, and restoring sex-hormone alterations in female APP/PS1 mice. We confirm that six-month-old APP/PS1 female mice develop a spontaneous gut dysbiosis, an estrobolome dysfunction, and lower oestrous cycle frequencies than their WT littermates. Soluble fiber intake modulates the gut microbiota composition and increases beta-glucuronidase activity in faecal samples associated with a restoration of estradiol and DHEA plasma levels. Additionally, consuming soluble fiber affects various bacterial metabolic pathways and enhances the production of butyrate and acetate. Improved memory and cognitive performance were also observed in female APP/PS1 mice that were fed fructans. Therefore, soluble fiber intake may represent a preventive strategy to mitigate the alterations associated with the onset of reproductive senescence and dementia in females.

**Supplementary Information:**

The online version contains supplementary material available at 10.1007/s10571-026-01745-3.

## Introduction

Alzheimer’s disease (AD) is a progressive neurodegenerative disorder with a long preclinical phase in which pathological changes develop years before the onset of cognitive symptoms (Breijyeh and Karaman 2020; Safiri et al. 2024; Prajapati et al. 2024b). Women are significantly impacted by AD, with two-thirds of AD patients being female (Alzheimer Association 2016; Scheyer et al. 2018). As neurodegenerative processes in the brain of AD patients may occur decades before clinical symptoms, in women, this may occur during their still active reproductive period, the perimenopause (Mosconi et al. 2017). However, the biological mechanisms that contribute to women’s increased vulnerability to AD remain largely unknown. On the one hand, the transition from perimenopause to menopause represents a midlife neuroendocrine transition state during which hormonal changes, particularly the decline of estrogen levels, may increase AD vulnerability (Mosconi 2026). A shorter reproductive lifespan and earlier onset of menopause are associated with an increased risk of developing AD (Gilsanz et al. 2018; Liao et al. 2023). Additionally, surgical removal of the ovaries before menopause can lead to neurological symptoms and cognitive impairments (Phung et al. 2010). On the other hand, sex hormones have been reported to influence gut microbiota composition, while the microbiota can also regulate sex hormone metabolism (Plottel and Blaser 2011). Alterations in the gut microbiota may be considered a contributing factor in the onset of neurodegenerative diseases, as patients with AD exhibit a progressive gut dysbiosis compared to healthy individuals of the same age (Cattaneo et al. 2017; Vogt et al. 2017; Zhuang et al. 2018; Haran et al. 2019; Li et al. 2019; Liu et al. 2019). This relationship between gut microbiota and estrogen levels is linked to the activity of the enzyme β-glucuronidase (Ervin et al. 2019) and to estrobolome function (Plottel and Blaser 2011; Catron et al. 2019). Recently, we demonstrated that gut dysbiosis in female mice with AD (APP/PS1) was associated with a dysfunctional estrobolome, characterised by lower faecal β-glucuronidase activity, reduced plasma estradiol levels, and increased faecal estradiol excretion. The dysfunction of the estrobolome in six-month-old female APP/PS1 mice was associated with behavioural alterations and earlier reproductive senescence (Romero-Flores et al. 2025). A promising strategy for restoring gut microbiota composition is the ingestion of dietary soluble fiber, specifically fructans, due to their potent prebiotic effects (Hughes et al. 2022). To further investigate the role of the gut microbiota in early stages of AD pathology, we used 4-month-old female APP/PS1 (TG) and wild-type (WT) mice fed a diet containing 5% fructans (F) for 2 months. This timeline aligns with a previous study showing that 4-month-old male APP/PS1 mice fed fructans for the same duration experienced improved cognitive function and reduced neuroinflammation (Cuervo-Zanatta et al. 2023). Moreover, six-month-old APP/PS1 female mice show significant memory deficits, gut dysbiosis (Cuervo-Zanatta et al. 2021; Romero-Flores et al. 2025), and exhibit a regular estrus cycle (Bahougne et al. 2019), making this stage comparable to premenopause in women. Our findings indicate that dietary fructan supplementation in six-month-old TG female mice modulates gut microbiota, restores plasma estradiol levels to those observed in WT mice, improves cognitive performance, reduces anxiety-like behaviour, and decreases the size of cortical Aβ plaques as well as intestinal Aβ immunoreactivity. Fructan intake regulates metabolic pathways associated with butyrate and acetate production, and overall brain and reproductive health. The current study supports the role of the estrobolome in female AD pathology and suggests that soluble fiber intake is an effective preventive strategy to protect cognitive function.

## Methods

### Animals

The mouse strain used, B6.Cg-Tg (APPswe, PSEN1dE9) 85Dbo/Mmjax, RRID: MMRRC_034832-JAX, was obtained from the Mutant Mouse Resource and Research Centre (MMRRC) at The Jackson Laboratory (USA). Female hemizygous Amyloid Precursor Protein/Presenilin 1 (APP/PS1: TG) and their wild-type (WT) littermates were used. All mice were housed individually with *ad libitum* access to food (Purina Rodent Chow 5001) and water until use. Animal management and health status were supervised by licensed veterinarian care and approved by the Bioethics Committee of the Center for Research and Advanced Studies of the National Polytechnic Institute (Protocol No. 235–16), under the Mexican official standard NOM-062-ZOO-1999 and the principles outlined in the 8th edition of the National Institute of Health (NIH) guide for the care and use of laboratory animals and endorsed by the Institute for Laboratory Animal Research, the Division on Earth and Life Studies and the National Research Council of the National Academies.

### Experimental Design

Four-month-old TG (*n* = 20) and WT (*n* = 20) female mice were used and randomly divided into a control (C; *n* = 10 each TG and WT) or soluble fiber-rich diet (F; *n* = 10 each TG and WT) group for two months. The sample size was determined a priori based on our previous published studies, which demonstrated that similar or smaller group sizes were sufficient to detect statistically significant differences between experimental groups (Cuervo-Zanatta et al. 2023; Prajapati et al. 2024a; Romero-Flores et al. 2025). To further support sample size adequacy, a post-hoc power analysis was performed using G*Power 3.1.9.7 software, indicating that our study was powered (1 – β = 0.80) to detect significant differences (α = 0.05).

**Fig. 1:**
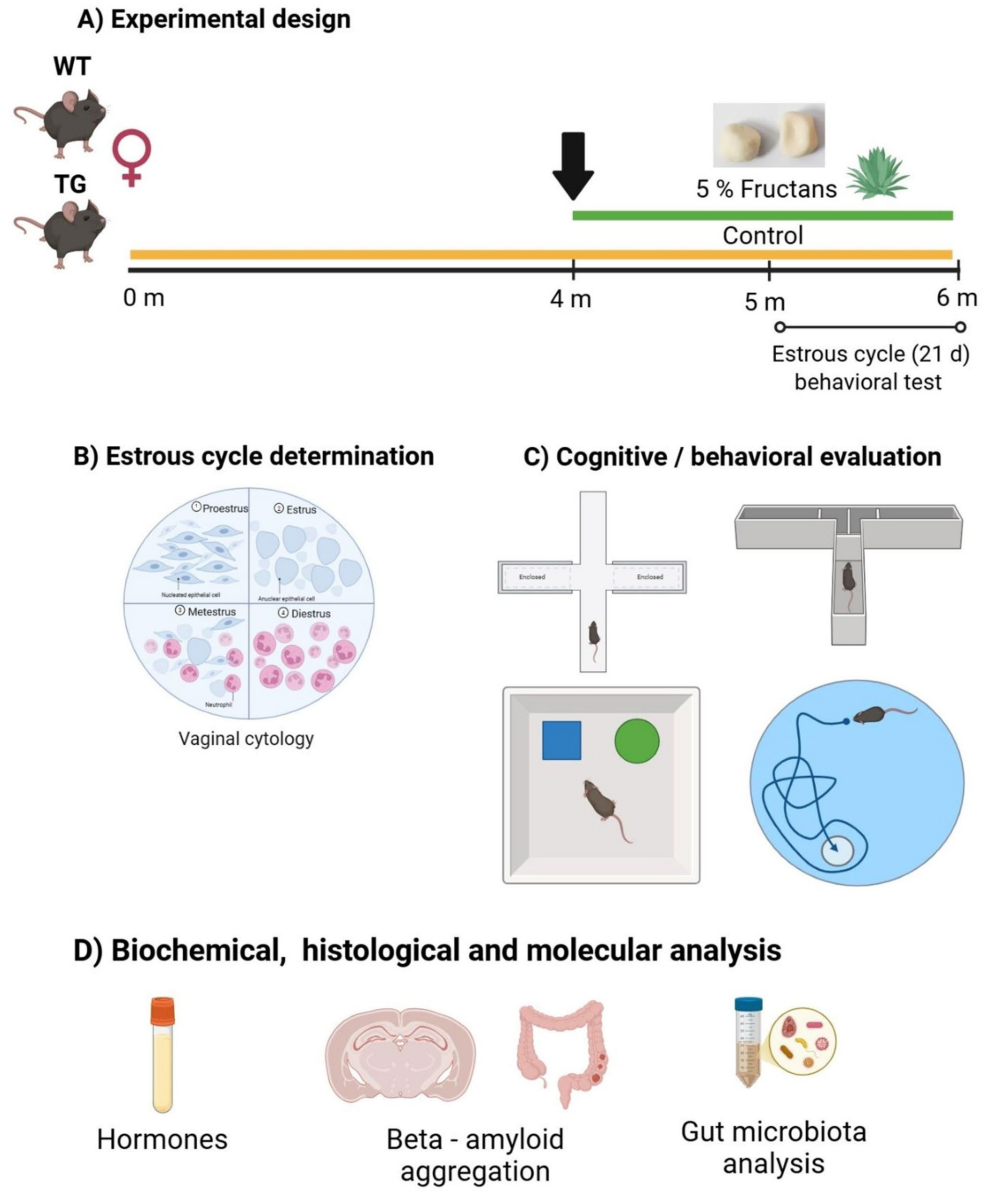
Schematic representation of experimental design. **(A)** Wild-type (WT, *n* = 20) and transgenic APP/PS1 (TG, *n* = 20) female mice were selected at four months of age and divided into two groups: Control (C) and 5% fructans (F) diet, followed by two months. **(B)** One month before the treatment completion, vaginal cytology was initiated to determine the estrous cycle for 21 days. **(C)** Behavioural tests were run to evaluate cognitive performance and anxiety during metestrus/diestrus. **(D)** Upon completion of the treatment, animals were euthanised, and plasma, brain, and faecal samples were collected for hormonal, amyloid aggregation, and gut microbiota analysis, respectively

C diet was prepared according to the American Institute of Nutrition (Reeves and Suppl 1993) with the following composition: Cornstarch (450.1 g/Kg), casein (140 g/Kg), maltodextrin (169.4 g/Kg), sucrose (100 g/ Kg), soy oil (40 g/Kg), cellulose (50 g/Kg), mineral mix (35 g/Kg), vitamin mix (10 g/Kg), l-cysteine (3 g/Kg), choline (2.5 g/Kg), tertiary butylhydroquinone (TBHQ; 0.01 g/Kg).F diet consists of adding 5%*Agave tequilana*´s derived fructans (Bustar Alimentos, S.A. de C.V.) to C diet resulting in the following composition: Cornstarch (400.1 g/Kg), casein (140 g/Kg), maltodextrin (169.4 g/Kg), sucrose (100 g/Kg), soy oil (40 g/Kg), cellulose (50 g/Kg), mineral mix (35 g/Kg), vitamin mix (10 g/Kg), l-cysteine (3 g/Kg), choline (2.5 g/Kg), tertiary butylhydroquinone (TBHQ; 0.01 g/Kg), and*A. tequilana*fructans (50 g/Kg). Both diets contain the same 3.90 kcal/g.A schematic representation of the experimental design is depicted in Fig. 1.

Body weight and food intake were monitored every two days. No significant differences in food intake or body weight change were observed at the end of treatment (data not shown). All authors were blinded to the experimental protocol while performing the experiments.

### Cognitive and Behavioural Evaluation

One week before finalising the dietary intervention, behavioural testing was performed. To reduce the potential impact of sex hormones on cognitive performance (Gurvich et al. 2018), only animals in the metestrus/diestrus stages of the estrous cycle were included in the cognitive assessment (Miller and Takahashi 2014). The estrous staging was performed through hematoxylin-eosin-stained vaginal cytology based on Wolcott et al. (2022) (Wolcott et al. 2022). Briefly, epithelial cells from the superficial vaginal cavity were exfoliated via phosphate-buffered saline (PBS). Samples were allowed to dry for 1 h at 37 °C on a glass slide, then exposed to the following reagents in the following order (3 min each): hematoxylin, water, eosin, water. Animals were habituated to the behavioural room two days before cognitive testing, which was run as follows:

**Elevated Plus Maze (EPM)** was used to evaluate anxiety-related behaviours as previously described (Cuervo-Zanatta et al. 2023). A 50 cm elevated plus-shaped maze (50 cm long × 10 cm wide) was used. Entries into the open/closed arms were recorded for five minutes, and the number of entries and the percentage of time spent in the arms were calculated.

**Novel Object Recognition (NOR) test.** Recognition and working memory were evaluated using the NOR test, as previously reported (Cuervo-Zanatta et al. 2023). Briefly, the NOR test was conducted in a 40 × 40 cm open-field arena and consisted of a habituation phase (5 min) followed by a training phase 24 h later. The “training trial” consisted of placing the rodents in the arena for 5 min, where two identical objects were positioned in adjacent corners at 10 cm from the walls. A “trial phase” was conducted 1.5 h after the training phase and consisted of placing the animals in the arena for 5 min in the presence of one familiar and one novel object (with different weights, textures, shapes, and colours). A discrimination index (DI) was calculated as: (time with novel object − time with familiar object) / (time with novel object + time with familiar object).

**T-maze (TM) test.** Working memory was evaluated by the T-maze as previously described (Cuervo-Zanatta et al. 2023). Briefly, a T-shaped maze apparatus with a starting arm (8.5 × 10.5 × 33.0 cm) and two choice arms (8.5 × 10.5 × 30.0 cm each, left and right) was used. The TM task consisted of a sample phase, during which animals were placed at the start arm and allowed to choose between the left and right arms, with a central divider inserted into the start arm to create a start box. Once animals had chosen an arm, they were confined by plastic doors for 30 s. After that, doors were reopened, and mice were allowed to return to the start arm, where they were removed and returned to their home cages. Two minutes later, animals were placed again to perform the task without the central divider. Once animals chose an arm (“Choice phase”), they were removed and placed in their home cages. Two hours later, the procedure was repeated. At least 3 trials per day for 2 days were conducted for each animal. The percentage of alternations between arms was calculated.

**Water Maze (WM) test.** Spatial memory was assessed by WM as previously described (Cuervo-Zanatta et al. 2023). The WM task was carried out in a 100 cm-diameter, 30 cm-high round pool partially filled with 26 °C white-painted water. WM task consisted of three phases: (1) Training phase: mice were deposited from three different cardinal points and allowed to swim to reach a visible platform for 60 s; in case the animals did not find the platform during this time, they were gently guided and left in the platform for 20 s; (2) Learning phase: mice were allowed to swim in order to find a hidden platform in a maximum time of 60 s for 12 consecutive trials (all in one-day), starting three non-consecutive times from each of the four different cardinal points; (3) Memory phase: After completion of the 12 trials, the platform was removed from the pool. Mice were released from the north side and allowed to swim to find the missing platform within 30 s. The time spent finding the platform (latency) and the number of crossings of the pool’s centre (quadrant crossings) were recorded.

All experiments were recorded with a Logitech C920 Pro webcam, connected to a computer running Logitech Capture 1.10.110 and Eniima (in-house software) to track the animal’s behaviour and trajectory.

### Sample Collection and Tissue Processing

Faecal samples were collected at the end of treatment. Briefly, animals were placed in a Super Mouse 750™ Micro-IsolatorR system cage (15 cm wide × 30 cm long × 17 cm high) for 1 h, which had been cleaned and disinfected. At least 100 mg of faeces pellets were collected per animal, immediately frozen, and stored at − 70 °C until use.

Once behavioural tests were finalised, animals were anaesthetized with pentobarbital (150 mg/Kg) during the metestrus/diestrus stage of the estrus cycle, and a blood sample was obtained from the retro-orbital sinus with a heparinised capillary and centrifuged at 2000 rpm for 15 min at 4 °C to obtain the plasma and frozen a -70 °C until use. The brains were immediately collected, and the hemispheres were divided for further analysis. The right hemisphere was post-fixed in 4% paraformaldehyde (PFA) for 72 h at 4 °C for immunohistochemistry (IHC), and the left hemisphere was immediately frozen (-70 °C) for Western blot. The proximal colon was dissected, washed in PBS 1X, fixed in 4% paraformaldehyde, and embedded in paraffin. Colon tissue was cut and post-fixed in 4% PFA for IHC. Brain sections were cryoprotected by immersion in 30% sucrose for 3 days. 40 μm thick coronal brain (Bregma − 1.70 mm to -2.30 mm AP) and 5 μm thick colon sections were obtained with a sliding microtome (Leica Jung histoslide 2000 R) and mounted on glass slides covered with silane (previously eliminated paraffin).

### Analysis of Hormone Levels in Plasma

An automated immunoassay (Immulite 1000 Progesterone kit, SIEMENS) was used to measure plasma progesterone levels. Estradiol, free testosterone, and dehydroepiandrosterone (DHEA) were analysed by an enzyme immunoassay (DRG Diagnostic) according to manufacturer’s instructions.

### β-glucuronidase Enzymatic Activity

β-glucuronidase activity was determined in faecal samples using a colourimetric method. Briefly, 300 µL of RIPA lysis buffer was added to 200 mg of the faecal sample, and the mixture was homogenised for 3 min. Samples were centrifuged at 14,000 rpm for 15 min at 4 °C, and the supernatant was recovered. The extracted enzyme fraction was used to measure β-glucuronidase activity according to the Enzymatic Assay of β-glucuronidase (EC 3.2.1.31) from *Escherichia coli* (Sigma-Aldrich). The enzymatic activity was expressed as units/g faecal.

### Immunohistochemistry and Western Blot Techniques

For Aβ-IHC, tissue sections were washed overnight in phosphate-buffered saline (PBS), then incubated in 89.8% formic acid (Baker) for 15 min, followed by 3 washes in PBS for 5 min each. Sections were incubated in 0.3% H2O2 + 10% methanol in PBS for 30 min, followed by washing in 0.1% tween in PBS (0.1% PBS-tween) and then incubated in 0.2% triton in PBS (PBS-triton) for 10 min, followed by incubation in 3% bovine serum albumin (BSA, Sigma) in 0.1% PBS-tween for 30 min. Thereafter, sections were incubated with an Aβ1–42 monoclonal antibody (Invitrogen, Cat. No. 44–344, 1:500) in 0.1% PBS-Tween overnight at 4 °C. After washing in 0.1% PBS-tween and PBS, sections were incubated with a secondary antibody (Anti-rabbit IgG, Jackson ImmunoResearch, 1:500) diluted in 1.5% horse serum in 0.1% PBS-tween for 2 h at room temperature. Subsequently, sections were washed and incubated with the avidin-biotin complex (ABC Kit; Vector Laboratories) in 0.1% PBS-tween for 1.5 h according to the manufacturer’s instructions. Finally, antibody binding was visualised with 0.025% 3,3’-diaminobenzidine (DAB Peroxidase Substrate Kit; Vector Laboratories) with 0.01% H2O2 as a catalytic agent. Sections were washed with PBS, dehydrated by serial dilutions in ethanol, and mounted on gelatin-coated slides with Eukit (Fluka). Control sections were processed without the primary antibody.

For Western blot experiments, the cortex was homogenised with RIPA lysis buffer and protease inhibitor. The homogenates were centrifuged at 12,000 rpm for 10 min at 4 °C. Total protein was quantified in the supernatant based on a BSA curve (0–2,000 µg/µL) read at 562 nm using the BCA method (Thermo Scientific-23228). The quantified supernatant was stored at -20 °C. The samples were loaded onto a 10% polyacrylamide gel, followed by electrophoresis at 100 V for 90 min and semi-wet transfer (Trans-Blot Turbo system, BioRad) at 25 V for 30 min on 0.2 μm PVDF membranes (Immun-Blot PVDF Membrane, BioRad-1620177). The membranes were incubated overnight at 4 °C with the anti-estrogen receptor alpha (ER-alpha antibody [E115], 1:100 Abcam-32063) and β-actin (1:500, Santa Cruz Biotechnology-SC4778) as a loading control. The membranes were subsequently incubated with the secondary antibody,, goat anti-mouse IgG (H + L)- HRP Conjugate (1:20,000; Bio-Rad 1721011). Membranes were developed with Thermo Scientific SuperSignal West Femto reagent (Thermo Scientific-34095) using the ChemiDoc™ XRS+ System (Bio-Rad). Image analysis was performed using ImageLab software. Assays were performed in duplicate for each sample, using independent transfers.

### Aβ Plaque Load Quantification

To detect Aβ plaques in the brain and Aβ-immunoreactivity (IR) in the colon, brightfield images were obtained by a 10x objective under a Nikon Eclipse 80i light microscope equipped with a Nikon DS-Ri1 camera. All images were taken at the same light exposure. Tissue background staining was subtracted from optical density values, and a threshold was determined by identifying the best signal-to-noise ratio using the wand tool in legacy mode (Leica Application Suite X). The number and area of Aβ plaques ≥ 100 µm2 were determined using the Fiji v. 1.46 (NIH, open access) plugin “measure” in the CA1 region of the dorsal hippocampus, cortex (secondary visual (V2L), secondary auditory (AuD) cortices), and layers I-VI from the entorhinal cortex in pixels-to-µm scaled images.

### Faecal Microbiota Analysis

Bacterial DNA was extracted from faeces from each animal using FavorPrep™ Stool DNA Isolation Mini Kit (Favorgen Biotech Corp, Cat. No: FASTI001-1) according to the manufacturer’s instructions. The purified DNA quantity was measured at 260/280 nm using a NanoDrop 2000 spectrophotometer (Thermo Scientific), and its quality was evaluated by 0.5% agarose gel electrophoresis. Genomic libraries of ~ 281 base pairs (bp) 16S rDNA third hypervariable region (V3) amplicons were generated for each experimental subject using specific V3-341F primers against V3 that also contained a different 12-bp Golay barcode (set of barcodes 1–100) complementary to position 340–356 of *Escherichia coli*16S rRNA molecule *rrnB* GeneBank J01859.1 and an overhang adapter specified by Ion Torrent, and V3-518R reverse primers complementary to position 517–533 of the same molecule (Corona-Cervantes et al. 2020). The amplicons of the V3 regions were generated by 1 × 50 µL polymerase chain reactions (PCR) containing 1 µL of 10 µM forward primer, 1 µL of 10 µM reverse primer (settings specified above), 0.25 µL of 5U/mL high thermostable DNA recombinant polymerase (Thermo Scientific No. EP0402), 1 µl of 10 mM dNTP mix (Thermo Scientific No. R0191), 4 µL of 25 mM MgCl2 (Thermo Scientific No. R0971), 5 µL of 10X taq buffer with KCl (100 mM Tris–HCl [pH 8.8 at 25°C], 500 mM KCl, and 0.8% volume/volume [% v/v] nonidet P40, Thermo Scientific No. B38), and 20 ng of genomic DNA in 37.75 µL of nuclease-free water (Thermo Scientific No. R0581) per reaction. Amplification was performed using the Applied Biosystems Veriti Thermal Cycler. Equivalent amounts of 1 to 100 barcoded amplicons (10 µg each) were combined for sequencing. Each mix was purified using an E-Gel iBase Power System (Invitrogen). The libraries’ size and concentration were determined using an Agilent 2100 Bioanalyzer, and libraries for each run were diluted to 26 pM prior to clonal amplification. Emulsion PCR was performed using the Ion OneTouch™ 200 Template Kit v2 DL (Life Technologies) according to the manufacturer’s instructions. Amplicon enrichment with ion spheres was done using Ion OneTouch ES. The sequencing was done using the Ion 316 Chip Kit v2 and the Ion Torrent Personal Genome Machine (PGM) System. After sequencing, reads were filtered by the PGM software to remove low-quality and polyclonal sequences. During this process, sequences matching the 3’ adapters were automatically trimmed and filtered. Ion Torrent PGM software, Torrent Suite v4.0.2, was used to demultiplex the sequenced data based on their barcodes. Poor-quality reads were eliminated from the datasets: quality score ≤ 20, homopolymers > 6, length < 200 nucleotides (nt), and errors in primers or barcodes. Filtered data were exported as FASTQ files. Demultiplexed sequencing data were analysed using the quantitative insights into microbial ecology (QIIME) software, version 2024.10. The filtered sequences were exported as FASTQ files, and amplicon sequence variants (ASVs) were identified from reads that met the quality criteria using the 2023.5 pipeline (Bolyen et al. 2019). Representative sequences were taxonomically assigned with the Greengenes2 2024.09 database. The analysis was performed using RStudio 2025.05.0 + 496; the data were imported into RStudio using the qiime2R 0.99.6 and phyloseq 1.50.0 packages to analyse microbial communities using relative abundance. The alpha diversity indices were computed from normalised ASV counts using the R package ‘Vegan’ to quantify diversity within the sample, using the Chao1, ACE, Shannon, Simpson, and Fisher indices. Chao1 and ACE index reflected the taxonomic richness of the community. The Shannon and Simpson indices reflect both species´ richness and evenness. The Fisher index reflected species diversity. The beta diversity indices were plotted as principal coordinates analysis (PCoA). Differential abundance analysis was performed with DESEq2 1.46.0, the data were analysed with tidyverse 2.0.0, and the figures were elaborated with ggplotify 0.1.2, and RColorBrewer 1.1-3. The Phylogenetic Investigation of Communities by Reconstruction of Unobserved States (PICRUSt2) pipeline version 2.0 was used to predict metagenomic function from 16 S rRNA gene data, implemented as a plugin in QIIME2 and executed in the same Ubuntu environment. The MetaCyc database was used to predict metabolic functions.

### Short-chain Fatty Acids Determination

Short-chain fatty acids (SCFAs) in faecal samples were analysed by high-performance liquid chromatography (HPLC; PerkinElmer Series N3896). The faecal samples were dried to constant weight and processed using the solid-phase extraction (SPE) method (Agilent Technologies 1260). At least 50 mg of dried faeces was suspended in 1 mL of deionised water by vigorous vortex mixing at maximum speed for 10 min. The suspension was centrifuged at 13,000 rpm for 10 min, and the supernatant was transferred to a fresh tube and passed through activated C-18 max 100 mg/ml GracePure™ Reversed-Phase SPE Columns according to the manufacturer´s instructions. SCFAs were eluted with 1 mL of filtered water (Nylon Syr Filter, 13 mm, 0.22 μm) and analysed by HPLC using 20:80 acetonitrile: NaH2PO4 (pH 2.2 with phosphoric acid, Sigma-Aldrich No. S8282-500G) as the mobile phase (De Baere et al. 2013). Standard curves were prepared for acetate (Sigma-Aldrich No. 45754-100ML-F), propionate (Sigma-Aldrich No. P1386-500ML), and butyrate (Sigma-Aldrich No. B103500-500ML).

### Statistical Analysis

Data are expressed as mean ± standard deviation (SD). Data were analysed by two-way analysis of variance (ANOVA) followed by Tukey´s post hoc test, except for SCFAs, which were analysed by one-way ANOVA followed by Tukey´s post hoc test. The analysis of microbial communities with relative abundance was done by metacore algorithms, and R (v3.6.0) in RStudio 2025.05.0 + 496 software. Differences in taxa enrichment and abundance of sequences were evidenced by DESeq2 and ANOVA. β-diversity was analysed using ANOSIM and ADONIS. Statistical analyses were performed with GraphPad Prism (v9.3.0). All results were considered statistically significant at *p* < 0.05. The relative abundances of MetaCyc pathways were expressed as mean ± SD and analysed using a two-group unequal variance test (Welch’s t-test), implemented in the open-source STAMP software (version 2.1.3). False Discovery Rate (FDR) correction was applied to adjust for multiple hypothesis testing when necessary. The significance level for multiple comparisons was set at 5%.

### Data and code availability

All sequencing data generated in this study are deposited at the NCBI Sequence Read Archive repository as bioproject PRJNA718599(https://www.ncbi.nlm.nih.gov/Traces/study/?acc=PRJNA718599*and* https://www.ncbi.nlm.nih.gov/sra/?term=PRJNA718599), and are publicly available as of the date of publication. Any additional information required to reanalyse the data reported in this paper is available from the lead contact upon considerable request.

## Results

### Dietary Soluble Fiber Initake Decreases Anxiety and Improves Cognitive Functions in TG Female Mice

**Fig. 2:**
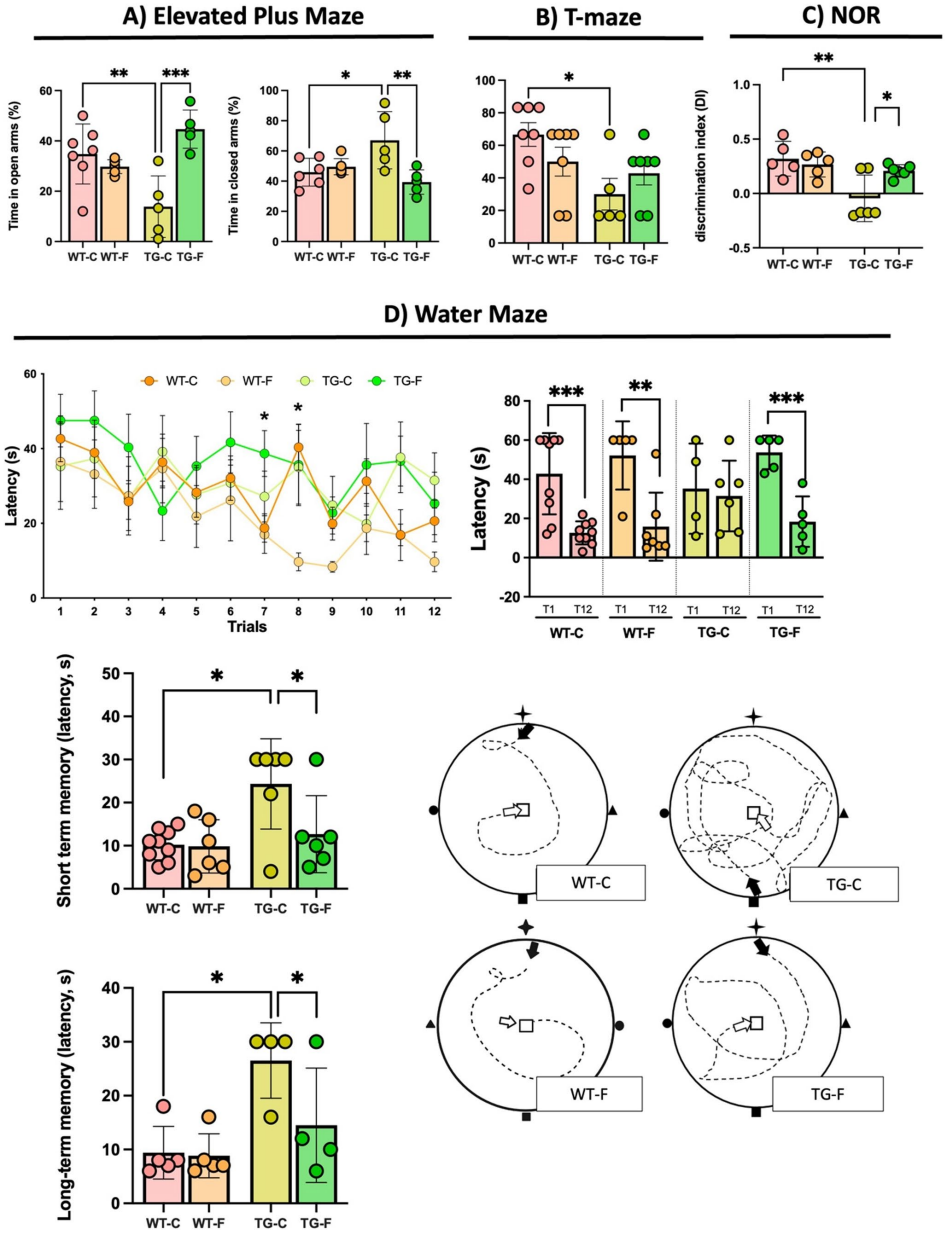
Effect of a dietary fiber intake on anxiety, working memory, recognition and spatial memory. **(A)** In the Elevated Plus Maze, TG-F mice spent more time in the open arms and less time in the closed arms compared with TG-C mice, indicating reduced anxiety-like behaviour. **(B)** Working memory was directly proportional to the percentage of spontaneous alterations in the T-maze test, and TG-C had the lowest percentage compared to WT-C. **(C)** The discrimination index, which is directly proportional to recognition memory, was significantly lower in TG-C mice compared with WT-C and TG-C mice. **(D)** Upper panels: Spatial learning in the water maze was inversely proportional to the latency to find the hidden platform (left panel). The x-axis represents 12 consecutive learning trials in the experiment. Learning performance was compared between trial 1 and trial 12 (right panel). Lower panels: Short-term and long-term memories improved after fiber supplementation in TG mice. Representative schemes of mice (WT-C, WT-F, TG-C and TG-F) trajectories (dotted line and black arrow) from the star point to the platform area (square and white arrow). For **(A)** WT-C *n* = 7, WT-F *n* = 6, TG-C *n* = 6, and TG-F *n* = 6. For **(B)** WT-C *n* = 7, WT-F *n* = 7, TG-C *n* = 5, and TG-F *n* = 7. For **(C)** WT-C *n* = 5, WT-F *n* = 5, TG-C *n* = 6, and TG-F *n* = 6. For **(D)** WT-C *n* = 9, WT-F *n* = 7, TG-C *n* = 6, and TG-F *n* = 5; Short-term memory: WT-C *n* = 9, WT-F *n* = 6, TG-C *n* = 6, and TG-F *n* = 6. Long-term memory: WT-C *n* = 5, WT-F *n* = 5, TG-C *n* = 4, and TG-F *n* = 4. The data are shown as mean ± standard deviation (SD). For (A), (B), (C), (D)- left panel, short and long-term memory- two-way ANOVA with Tukey’s post hoc correction was used. For (D- right panel), Student’s *t*-test was used. Statistical significances are shown as *p < 0.05, **p < 0.01, ***p < 0.001, ****p < 0.0001

We evaluated anxiety by assessing the percentage of time spent in the open and closed arms of the EPM. There was a significant interaction between genotype and diet [F(1,19) = 20.02, *p* = 0.0003] in the open arms and a significant effect of diet [F(1,19) = 10.38, *p* = 0.004], as TG-C females spent less time in the open arms than WT-C (*p* = 0.006) and TG-F mice (*p* = 0.0003). In the closed arms, there was a significant interaction between genotype and diet [F(1,18) = 10.59, *p* = 0.004] with a significant effect of diet [F(1,18) = 6.26, *p* = 0.02], as TG-C females spent more time in the closed arms than WT-C and TG-F mice (*p* = 0.02 and *p* = 0.005, respectively) (Fig. 2A). Therefore, a two-month dietary fiber intake reduced anxiety in TG female mice.

Short-term working memory, recognition, and spatial memory were evaluated by the T-maze test (TM), the novel object recognition test (NOR), and the water-maze test (WM), respectively. In the TM, genotype had a significant effect [F(1,22) = 7.01, *p* = 0.01]: TG-C mice had a lower percentage of spontaneous alternations than WT-C (*p* = 0.03), while TG-F mice showed values similar to those of WT-C and WT-F (Fig. 2B). We determined the discrimination index (DI) in the NOR test. There was a significant interaction between genotype and diet [F(1,18) = 5.68, *p* = 0.02] and a significant effect of genotype in DI [F(1,18) = 10.92, *p* = 0.003], as TG-C mice showed a lower DI than WT-C (*p* = 0.004) and TG-F (*p* = 0.04) (Fig. 2C). In the WM, the latencies to find the platform during the learning phase were decreased by the consecutive trials in all groups, but they showed a significant difference on Day 7 (WT-C < TG-F, *p* = 0.04) and Day 8 (WT-F < WT-C, *p* = 0.03). To evaluate effective learning, we compared the latencies to find the hidden platform between trial 1 (T1) and T12. Statistically significant differences were obtained between T1 and T12 in WT-C (t = 4.201, df = 16, *p* = 0.0007), WT-F (t = 3.565, df = 10, *p* = 0.005) and TG-F (t = 5.126, df = 8, *p* = 0.0009), but not in TG-C mice, indicating impaired learning in TG-C female mice. After completing the twelve consecutive trials, short-term memory was evaluated by removing the hidden platform. There was a significant effect of genotype [F(1,24) = 8.86, *p* = 0.006], as TG-C female mice spent more time trying to find the place where the platform was located compared to WT-C and TG-F female mice (both *p* = 0.05). Long-term memory was evaluated twenty-four hours after the last trial. There was a significant effect of genotype [F(1,15) = 10.93, *p* = 0.004] as higher latencies to find the place where the platform was located were observed in TG-C compared to WT-C and TG-F (both, *p* = 0.05) (Fig. 2D). Thus, a two-month F intake prevented hippocampal and cortical-related cognitive impairments in female TG-F mice.

### Dietary Soluble Fiber Effectively Restores the Estrobolome Function and Regulates the Estrous Cycle in TG Female Mice

**Fig. 3:**
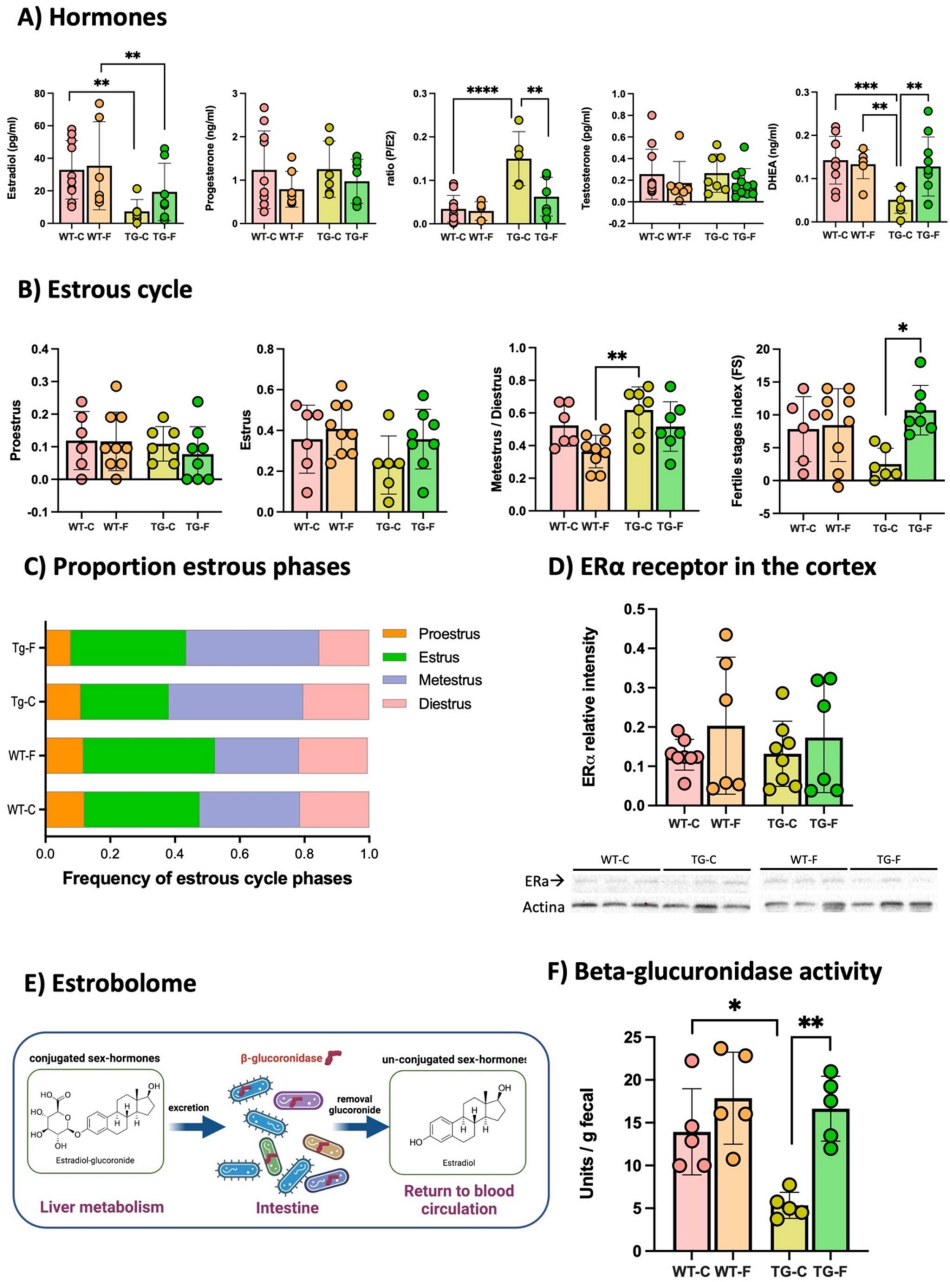
Influence of dietary fiber intake on hormonal levels, estrous cycle and beta-glucuronidase enzymatic activity. **(A)** Estradiol levels were lower in TG-C females compared to WT-C, and in TG-F compared to WT-F groups. The ratio (P/E2) was significantly higher in TG-C than in WT-C and TG-F. Progesterone and testosterone showed similar values in all groups. DHEA levels were significantly lower in TG-C than in WT-C and WT-F. DHEA levels were higher in TG-F than in TG-C. **(B)** Proestrus and estrus stages showed a similar frequency in all groups. Metestrus/diestrus frequencies were increased in TG-C compared to WT-F. The Fertile Stage index (FS) increased in TG-F compared to TG-C. **(C) Schematic** representation of the frequency of estrous stages in twenty-one days. **(D)** ERα receptor in the cortex showed a similar relative expression level in all the groups. **(E)** Estrobolome function: Phase II metabolism of estradiol takes place in the liver, as UDP-glucuronosyltransferase enzymes (UGTs) add on a glucuronic acid molecule to estrogens to inactivate them. In the gut, some bacteria can metabolise estrogens via the β-glucuronidase enzyme, releasing active estrogens back into the circulation. **(F)** β-glucuronidase activity was lower in TG-C compared to WT-C mice. However, dietary fiber intake significantly increases β-glucuronidase activity in TG-F compared to TG-C. For **A) estradiol**: WT-C *n* = 10, WT-F *n* = 6, TG-C *n* = 6, and TG-F *n* = 7; **progesterone**: WT-C *n* = 10, WT-F *n* = 7, TG-C *n* = 6 and TG-F *n* = 7; **testosterone**: WT-C *n* = 10, WT-F *n* = 7, TG-C *n* = 7 and TG-F *n* = 10; **DHEA**: WT-C *n* = 10, WT-F *n* = 7, TG-C *n* = 7, and TG-F *n* = 10. For **B-C)** WT-C *n* = 6, WT-F *n* = 9, TG-C *n* = 7, and TG-F *n* = 8. For **D)** WT-C *n* = 6, WT-F *n* = 6, TG-C *n* = 6, and TG-F *n* = 6. For **F)** WT-C *n* = 5, WT-F *n* = 5, TG-C *n* = 5, and TG-F *n* = 5. Outliers (identified using the ROUT method, Q = 1%) were removed from Figs. 2A-C. Data is shown as mean ± Standard Deviation (SD). Statistical analyses were performed by two-way ANOVA followed by Tukey’s post hoc test. Statistical significances are shown as *p < 0.05, **p < 0.01, ***p < 0.001, ****p < 0.0001.

We determined the levels of estradiol, progesterone, testosterone, and DHEA in plasma samples from six-month-old WT and TG female mice fed a fructan diet or a control diet. There was a significant effect of genotype [F(1,28) = 7.82, *p* = 0.009] in estradiol plasma levels, as female TG-C mice had lower estradiol levels than WT-C (*p* = 0.009), and between WT-F and TG-F (*p* = 0.009). Progesterone and Testosterone showed similar values in all groups. However, the ratio (P / E2) showed a significant effect of genotype [F(1,25) = 23.81, *p* = 0.0001] and diet [F(1,25) = 9.11, *p* = 0.005], with a higher ratio in TG-C compared to WT-C (*p* = 0.0001) and TG-F (*p* = 0.003). DHEA levels had a positive interaction between genotype and diet [F(1,29) = 6.87, *p* = 0.01] and a significant effect of genotype [F(1,29) = 9.13, *p* = 0.005) and diet [F(1,29) = 6.97, *p* = 0.01)], as TG-C females had lower DHEA levels than WT-C and WT-F (*p* = 0.0003 and *p* = 0.001, respectively), and compared to TG-F (*p* = 0.001) (Fig. 3A). These data indicate that the intake of F over a two-month period effectively restored the levels of estradiol and DHEA in TG-F female mice to levels nearly equivalent to those of the WT-C group.

We analysed the frequency of the estrous cycle stages over 21 consecutive days. No significant differences were observed in the frequency of proestrus or estrus stages among groups. However, TG-C females showed a tendency toward fewer days in estrus than the other experimental groups, with significant effects of genotype [F(1,25) = 6.55, *p* = 0.01] and diet [F(1,25) = 7.29, *p* = 0.01]. A higher frequency of metestrus/diestrus stages was observed in TG-C than WT-F female mice (*p* = 0.003). We calculated the Fertile Stages index (FS) by calculating the number of days on each estrous stage (fertile stages: estrus = 1 and proestrus = 2; infertile stages: metestrus = 0 and diestrus = -1) during a 21-day period. Diet has a significant effect on FS [F(1,24) = 6.40, *p* = 0.01] as TG-F females had higher scores than TG-C mice (*p* = 0.03) (Fig. 3B). A schematic representation of the frequency of the estrous cycle in twenty-one days is shown in Fig. 3C.

ER-alpha receptor expression was determined in the cortex. No significant differences were observed among groups (Fig. 3D), despite slightly higher mean values in WT-F mice.

We evaluated the estrobolome function (Fig. 3E) by measuring β-glucuronidase activity in faecal samples collected at the end of the experiment (weeks 7–8 of treatment). There was a positive interaction of diet [F(1,16) = 16.30, *p* < 0.001] and genotype [F(1,16) = 6.78, *p* = 0.019] on β-glucuronidase activity, with significant differences between WT-C and TG-C (*p* = 0.0316) and between TG-C and TG-F (*p* = 0.003), indicating that TG-C females have a reduced β-glucuronidase activity, but dietary fiber intake was able to increase the β-glucuronidase activity in TG-F female mice (Fig. 3F). This data indicates that F intake over a period of two months effectively restores the b-β-glucuronidase activity and the estrobolome function in TG female mice, with important effects in P/E2 ratio and DHEA levels.

An increased Aβ load during aging is associated with memory impairments (Lim et al. 2014). As we observed preserved cognitive function in TG-F mice, we evaluated whether this neuroprotective effect was associated with changes in Aβ aggregation.

### Amyloid-beta Aggregation in the Brain and the Intestine of TG Female Mice

**Fig. 4:**
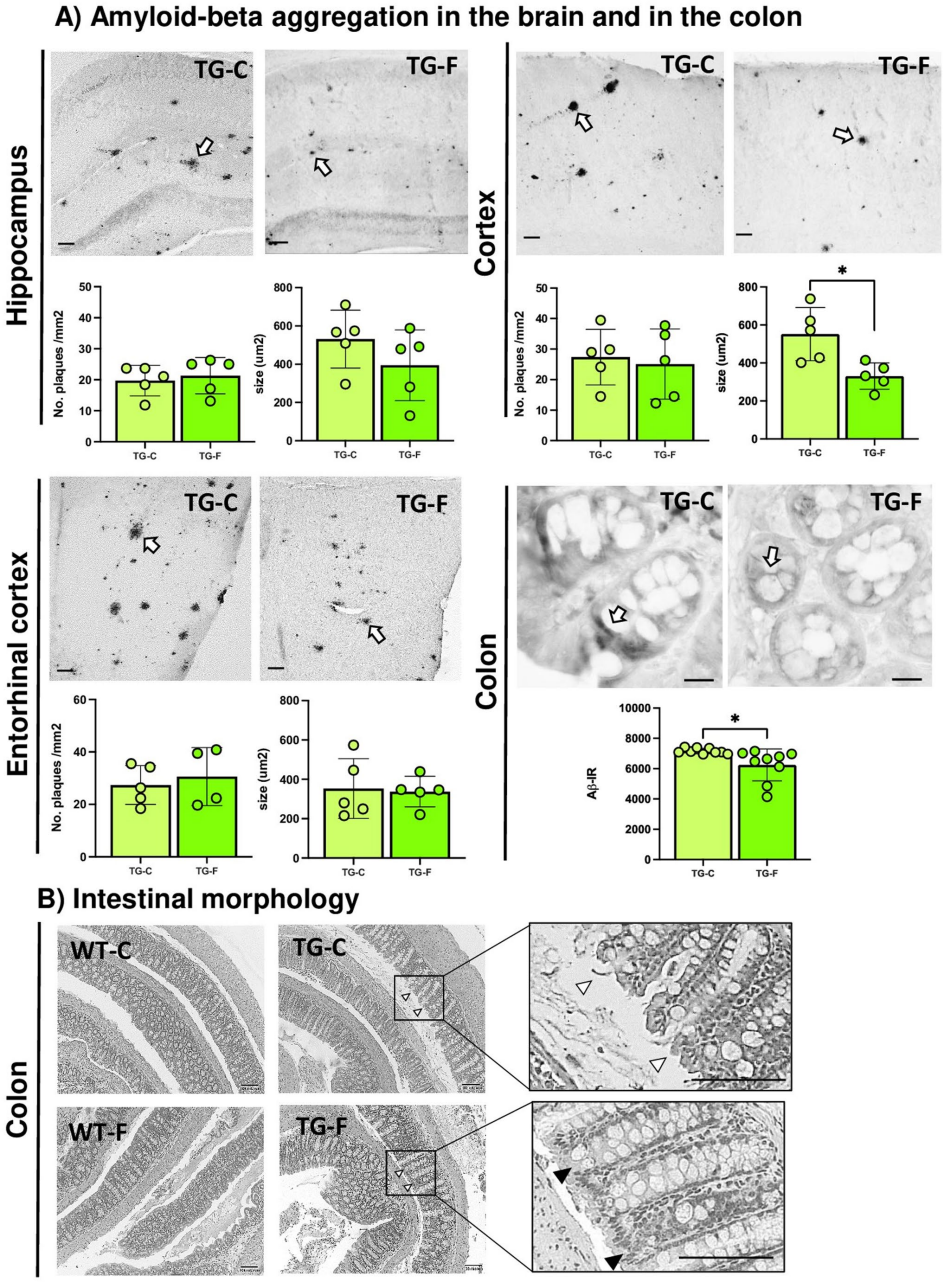
Aβ aggregation in the brain, the colon, and intestinal morphology. **(A)** Representative images of Aβ plaques in the CA1 region of the dorsal hippocampus, cortex [secondary visual (V2L) and secondary auditory (AuD) cortices] and layers I-VI of the entorhinal cortex in TG-C and TG-F female mice. Amyloid plaques are pointed by white arrows. No significant differences in the number of Aβ plaques were observed in any of the brain regions evaluated. In the cortex, the size of Aβ plaques decreased significantly in TG-F than in TG-C. In the colon, we observed positive Aβ immunoreactivity (Aβ-IR) in TG mice. TG-F female mice showed a lower Aβ-IR compared to TG-C females. Scale bar 100 μm. **(B)** Representative images of cross-sectional colon sections of WT-C, WT-F, TG-C and TG-F. White arrowheads indicate epithelial layer disruption in TG-C mice, and an apparent restoration in TG-F mice (black arrowheads). Scale bar 100 μm. For **A)** hippocampus, cortex and entorhinal cortex TG-C *n* = 5 and TG-F *n* = 5; colon TG-C *n* = 9 and TG-F *n* = 9. The data is shown as mean ± SD. Statistical analyses were performed by Student’s *t*-test. Statistical significances are shown as *p < 0.05, **p < 0.01, ***p < 0.001, ****p < 0.0001.

We evaluated the number and size of Aβ plaques in the CA1 region of the hippocampus, cortex, and the entorhinal cortex of TG-C and TG-F female mice. There were no differences in the number of Aβ plaques in any of the regions examined. However, the size of Aβ plaques in the cortex was smaller in TG-F female mice (Fig. 4A).

We also analysed the presence of Aβ aggregates in the colon. We observed clear Aβ-positive staining (Aβ-IR) in TG female mice, mainly in goblet cells. TG-F female mice showed lighter Aβ-IR that was significantly lower compared to TG-C (t = 2.591, df = 16, *p* = 0.01) (Fig. 4A, lower right panel). We previously observed a distinct intestinal morphology in six-month-old male APP/PS1 mice (Cuervo-Zanatta et al. 2023). Therefore, we conducted a histological evaluation of the distal colon in the four female groups. There were no apparent morphological differences in the colon of WT-C and WT-F female mice. However, we observed that the intestinal epithelial layer was disrupted in TG-C, potentially leading to a dysfunctional mucosal barrier. A restored morphology was apparent in TG-F female mice (Fig. 4B).

### Dietary Soluble Fiber Intake Alters the Gut Microbiota Profile in Female TG Mice

**Fig. 5:**
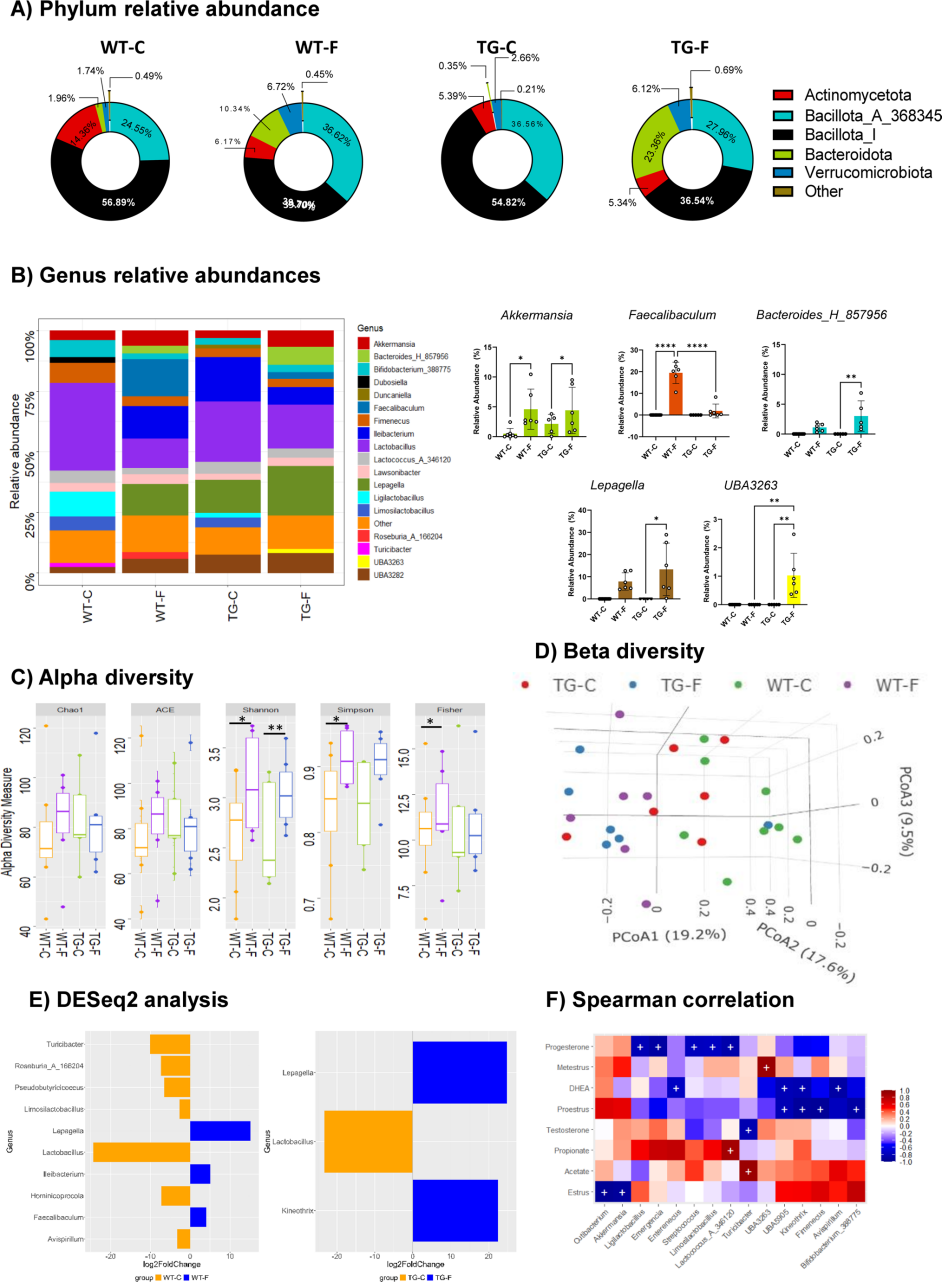
Composition of the gut microbiota in fecal samples. **(A)** Relative abundance of phylum level (with relative abundance over 1%). The number on the pie chart indicates the percentage of relative abundance. The colour of each tag on the right side indicates the different phylum found. Others refer to Phyla with relative abundance < 0.1% (Supplementary Material Table S1). **(B)** Relative abundance genus level (with relative abundance over 1%). The colour of each tag on the right side indicates the different genus found (Supplementary Material Table S2). The graphs on the right side show the relative abundance of prominent bacterial genera that were decreased or increased in WT and TG female mice after a fiber-rich diet. **(C)** The alpha diversity of faecal microbiome from mice, measured using the Chao1, ACE, Shannon, Simpson and Fisher indices. The Y-axis indicates alpha diversity, and the X-axis shows the experimental groups (Supplementary Material Table S3). The Shannon index and species richness were significantly higher in the fructan-treated groups (WT-F and TG-F) than in the control groups (WT-C and TG-C). **(D)** Beta diversity analyses (difference in global microbiota composition between groups) plotted as principal coordinates (PCoA). Percentages on the axes of PCoA plots indicated the proportion of variation explained by the axis. Each point in the graph represents a sample. **(E)** Differential abundance analysis of bacterial genera with DESeq2. Log2 Fold Change is shown by horizontal bars. **(F)** Spearman correlation analysis between gut microbiota and measured biological variables. Red squares indicate significant positive correlation, and blue squares indicate significant negative correlation. For **A-F)** WT-C *n* = 8, WT-F *n* = 6, TG-C *n* = 6, and TG-F *n* = 6. For **A-B)** data are shown as the mean. For **A-D)** Comparisons were performed using two-way ANOVA by Tukey’s post hoc. Statistical significances are shown as *p < 0.05, **p < 0.01, ***p < 0.001, ****p < 0.0001

According to the International Scientific Association for Probiotics and Prebiotics (ISAPP), prebiotics are substrates *selectively* utilised by host microorganisms, conferring health benefits (Gibson et al. 2017). Fructans are prebiotics (Hughes et al. 2022), meaning the host does not utilise them; instead, they are digested by some gut bacteria. Therefore, the effects of fructans in the current protocol rely on the gut bacterial fermentation and remodelling of gut microbiota communities. We analysed the microbiota profile at the end of 2 months of F treatment in WT and TG female mice and compared it with that of the control groups. At the phylum level, there were statistical differences among groups: Actinomycetota exhibited a higher relative abundance in WT-C compared to WT-F (14.36% vs. 6.17%) (*p* = 0.0049) and TG-C (14.36%) (*p* < 0.0001) female mice. Similarly, Bacillota I was higher in WT-C (56.89%) than in WT-F (39.70%) (*p* = 0.0003) and in TG-C (54.82%) than in TG-F (36.54%) (*p* = 0.001). Interestingly, the intake of dietary soluble fiber increased the relative abundance of Bacteroidota, with significant differences in the following groups: WT-F > WT-C (10.34% 1 > 1.96%)(*p* = 0.0311) and TG-F *>* TG-C (23.36% > 0.35%) (*p* < 0.0014).In addition, Verrucomicrobiota was higher in TG-F vs. TG-C (6.12% vs. 2.66%) (*p* = 0.0334) (Fig. 5A, and statistical analysis in Supplementary Material Table S1).

At the genus level, F intake in TG and WT female mice induced a higher abundance of specific taxa, such as *Akkermansia* [WT-F 4.60% vs. WT-C 0.5%, *p* = 0-0195; TG-F, 4.40% vs. TG-C 2.13% (*p* = 0.0338); and *Faecalibaculum* [WT-F vs. WT-C (19.44% vs. 0%) (*p* < 0.0001); TG-C vs. TG-F (1.88% vs. 0%) *p* < 0.0392). Moreover, female TG-F mice had some specific bacteria that were absent in TG-C mice, such as *Bacteroides_H_857956* (3.02%), *Lapegella* (13.32%), and UBA3263 (1.03%) (Fig. 5B left panel; and statistical analysis in Supplementary Material Table S2). On the other hand, TG-C female mice showed higher abundances of specific taxa that were absent or lower in the other experimental groups, such as *Duncaniella* (2%), which was higher than in WT-C (0%) (*p* = 0.0022). Interesting, some specific taxa decreased after F intake, such as *Limosilactobacillus* (WT-C, 4.11% vs. WT-F, 0%; *p* < 0.0001), *Bifidobacterium_388775* (WT-C, 5% vs. WT-F, 1.33%, *p* < 0.0001), *Fimenecus* (WT-C, 5.87% vs. WT-F, 0.66%, *p* < 0.0001), *Lactobacillus* (WT-C, 25.79% vs. WT-F, 7.37%, *p* < 0.0001) and *Lactococus_A_346120* (WT-C, 3.63% vs. WT-F, 1.56%, *p* = 0.0001)( Supplementary Material Table S2).

Alpha diversity was estimated using the Chao1, ACE, Shannon, Simpson, and Fisher indexes. The Shannon index showed a higher alpha diversity in F-treated groups compared to the C groups: WT-F vs. WT-C (3.13%, 2.16%) (*p* = 0.0499) and TG-F vs. TG-C (3.05%, 1.70%) (*p* = 0.0041). Simpson and Fisher indices also show a higher alpha diversity in WT-F groups compared to WT-C mice [(0.95%, 0.83%) (*p* = 0.0191); [(14.64%, 9.15%) (*p* = 0.0181)], respectively, indicating an increased microbial diversity (Fig. 5C and statistical analysis in Supplementary Material Table S3). Clustering the bacterial communities using principal coordinates analysis (PCoA) revealed segregation between the groups, with a notable distance between the F-treatment (WT-F and TG-F) and the C groups (WT-C and TG-C). This indicates that the bacterial composition was similar within each group, but distinct between groups (Fig. 5D). The result of DESeq2 analysis revealed significantly different bacterial abundance between F and C groups, as *Lactobacillus* was significantly enriched in the WT-C group, whilst *Lepagella* was enriched in the WT-F female mice. DESeq2 analysis also shows that the TG-C female group was characterised by enrichment of *Lactobacillus*, and the TG-F group mice were characterised by *Lapagella* and *Kineothrix* (Fig. 5E). Spearman correlation analysis was used to assess statistically significant correlations between taxa and the measured features in the multivariate analysis. *Ligilactobacillus*,* Emergencia*,* Streptococcus*,* Limosilactobacillus*, and *Lactococus_A_346120 were* negatively associated with plasma progesterone levels. *Enterenecus*,* UBA5905*,* Kineothrix*, and *Avispirillum were* negatively associated with DHEA levels in plasma. *Turicibacter* was also negatively associated with testosterone levels.*UBA3263* was positively associated with metestrus stages; *UBA5905*,* Kineothrix*,* Fimenecus*,* and Bifidobacterium_388775* were negatively associated with proestrus; and C*utibacterium* and *Akkermansia* were negatively associated with estrus stages. *Lactococcus_A346120* and *Turicibacter* were positively associated with propionate and acetate production, respectively (Fig. 5F).

### Dietary Soluble Fiber Intake Restores Short-chain Fatty Acids Production and Modulates Predicted Metabolic Pathways

**Fig. 6:**
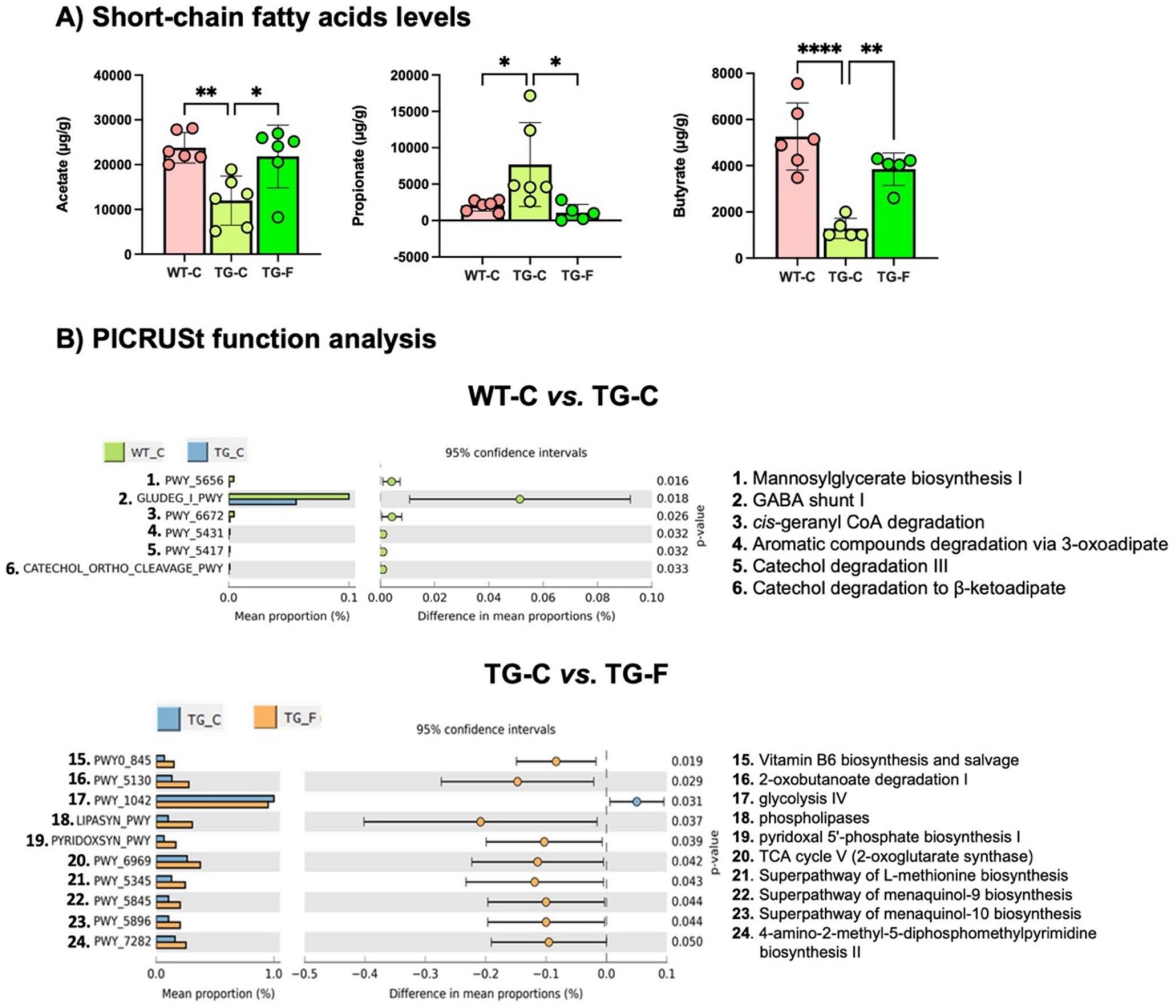
Fatty acids and predicted metabolic pathway analysis from gut microbiota. (A) Butyrate and acetate levels increased in TG-F, and propionate decreased compared to TG-C mice. (B) Bar plots showing MetaCyc pathways classification of metabolic pathways: WT-C vs. TG-C and TG-C vs. TG-F. For (A) WT-C *n* = 6, TG-C *n* = 6, and TG-F *n* = 6. For (B) WT-C *n* = 8, TG-C *n* = 6 and TG-F *n* = 6. For A) Comparisons were performed using One-way ANOVA followed by Tukey’s post hoc test. Statistical significances are shown as *p < 0.05, **p < 0.01, ***p < 0.001, ****p < 0.0001. Metabolite screening criteria: *p* < 0.05, comparisons were performed using Welch t-test applied False Discovery Rate (FDR) correction

It is known that the health benefits of prebiotics result from changes in the microbiota profile and microbial metabolites, such as SCFAs (Hughes et al. 2022). Therefore, we quantified the concentration of acetate, butyrate and propionate in faecal samples. Butyrate levels were significantly different between groups [F(2, 13) = 2.03, *p* = 0.0001], with lower levels in TG-C than in WT-C (*p* = 0.0001) and TG-F (*p* = 0.004). Acetate levels differed significantly among groups [F(2,15) = 0.31, *p* = 0.004], with lower values in TG-C than in WT-C (*p* = 0.005) and TG-F (*p* = 0.02). Propionate showed significant differences among groups [F(2,14) = 1.91, *p* = 0.01], with higher levels in TG-C than in WT-C (*p* = 0.02) and TG-F (*p* = 0.03) (Fig. 6A). This data demonstrates the potential of fructans to induce acetate and butyrate production while decreasing propionate. Functional prediction of metabolic pathways analysis (Fig. 6B and Supplementary Figure S1) was performed across all experimental groups. TG-C mice showed a decrease in the expression of the GABA shunt I pathway compared to WT-C mice. On the other hand, TG-F female mice show an increase in pathways related to Vitamin B6 biosynthesis, 2-oxobutanoate degradation I, phospholipases, pyridoxal 5’-phosphate biosynthesis I, TCA cycle, of L-methionine biosynthesis, menaquinol-9 and menaquinol-10 biosynthesis, and 4-amino-2-methyl-5-diphosphomethylpyrimidin biosynthesis II compared to TG-C female mice (Fig. 6B). Other statistical differences observed between WT-C vs. WT-F and WT-F vs. TG-F are shown in Supplementary Figure S1.

## Discussion

The primary objective of this study was to investigate the effects of soluble fiber consumption on the pathology of female mice with AD. We found that after a two-month intervention involving a diet high in soluble fiber, there were notable improvements in cognitive function (see Fig. 2), as well as increases in plasma estradiol and DHEA levels (see Fig. 3), along with a higher fertile stage index (see Fig. 3) in the treated TG female mice. In addition, our current data confirms previous reports showing that six-month-old APP/PS1 female mice had an estrobolome dysfunction (assessed by faecal β-glucuronidase activity and plasma estradiol levels) compared to age-matched WT females, associated with a depletion of β-glucuronidase-carrying bacteria that led to a higher estradiol excretion, and an earlier reproductive senescence (Romero-Flores et al. 2025). The interplay between the gut microbiota and circulating levels of sex hormones has been previously reported (Santos-Marcos et al. 2018). This is crucial for Alzheimer’s disease research, as patients develop significant gut dysbiosis as the disease progresses (Li et al. 2019; Liu et al. 2019). Hence, modulating the gut microbiota may help to influence the estrobolome and AD disease progression in females.

Targeting gut dysbiosis through dietary interventions has emerged as an attractive strategy for preventing or alleviating AD, as diet plays a crucial role in modulating the composition of gut microbiota (Bourdeau-Julien et al. 2023). The MIND diet in AD patients (Morris et al. 2015) or in people at high risk of developing dementia (Kivipelto et al. 2013; Morris et al. 2015) has shown promising results (Kivipelto et al. 2013; Rosenberg et al. 2020; Pöyhönen et al. 2025). Additionally, the intake of prebiotics (i.e., fiber rich diets) enhances cognition (Desmedt et al. 2019; Romo-Araiza and Ibarra 2020; Cuervo-Zanatta et al. 2023) and reduces anxiety by influencing the composition of the gut microbiota in mouse models (Gareau et al. 2011; Bercik et al. 2011; Syeda et al. 2018). However, interventions with soluble fiber may have differential health impacts depending on an individual’s initial gut microbiota profile and the underlying cause of the disease. High fiber intake has been associated with lengthening of the menstrual cycle and the follicular phase in healthy young women (Goldin et al. 1994), strongly suggesting that fiber intake modulates hormone levels. Soluble fiber, which dissolves in water, is derived from the inner flesh of plants. In contrast, insoluble fiber does not dissolve in water and comes from the outer skin of plants and typically cannot be fermented by bacteria in the colon (DeVries 2003). Here, we use a single source of soluble fiber (5% fructans from *Agave tequilana*) in the diet for 2 months in 6-month-old female mice, a reproductive life stage that can be comparable to premenopause in women (Finch et al. 1984; Bahougne et al. 2019). Moreover, APP/PS1 mice not only present cognitive dysfunction but also develop gut dysbiosis with aging (Harach et al. 2017; Zhang et al. 2017; Sun et al. 2019).

Our current data show that two months of soluble fiber intake restores estradiol levels and the progesterone to estradiol ratio (P/E2) in TG-F female mice approaching those of WT mice, whereas DHEA levels increased in TG females receiving soluble fiber. It is widely acknowledged that estradiol has beneficial effects on cognition. Estradiol replacement therapy restores spine density in ovariectomized (OVX) mice and improves short-term working memory in middle-aged rats with follicle depletion (Koebele et al. 2020). Additionally, estradiol administration enhances spatial memory in OVX-5xFAD female mice (Kim et al. 2022). Conversely, estrogen depletion following OVX impairs recognition memory (Wallace et al. 2006) and spatial learning in mice (McLaughlin et al. 2008). On the other hand, DHEA, a neuroactive steroid hormone secreted by the reticular zone of the adrenal glands, plays an important role in modulating synaptic connectivity (Friess et al. 2000). AD patients show lower levels of DHEA in the brain than matched control subjects (Weill-Engerer et al. 2002). Additionally, in cultured rat neuroblastoma cells, DHEA has been shown to reduce neurite atrophy induced by Aβ25–35 (El Bitar et al. 2014). The increased concentration of DHEA and estradiol in soluble fiber-treated TG mice (Fig. 3A) may be linked to the favorable growth adaptation of certain β-glucuronidase expressing bacteria resulting from the fermentation of fructans (see below, and Figs. 3E), as β-glucuronidase plays an important role in the deconjugation of estrogens and *other hormones*, thereby enabling their active forms to enter the bloodstream (Ervin et al. 2019).

To determine whether the cognitive improvements in TG-F female mice were associated with estrogen receptor (ER) expression in the brain, we assessed ER-alpha levels in the cortex. There were no significant differences in the relative intensity of ERα among the groups, although a slightly higher expression was observed in WT-F female mice. ERα and ERβ receptors are biological substrates for estrogen, but membrane-localised ERs have recently been linked to cognitive and social behaviours influenced by estradiol (Taxier et al. 2020). Therefore, further studies should investigate regional variations—specifically between the hippocampus and cortex—in the expression of ERα, ERβ, and membrane-localised ER in fiber-treated female TG mice.

Evaluation of the mice’s estrous cycle indicates stage differences in TG-C female mice. An increased metestrus/diestrus was observed in TG-C compared to WT-F mice (Fig. 3), similar to previously reported (Romero-Flores et al. 2025). The increased number of metestrus/diestrus stages and the reduced levels of estradiol in plasma in six-month-old TG-C mice may suggest an earlier perimenopause transition compared to age-matched WT females. Reproductive senescence in mice begins around 9–12 months of age, a period characterised by endocrine alterations (Finch et al. 1984; Cruz et al. 2017), including irregular estrous cyclicity and altered ovarian and hormonal oscillations that culminate in a persistent diestrus-like state (Bacelar et al. 2023). Soluble fiber intake in six-month old TG female mice was associated with a restoration of estradiol plasma levels, reduced metestrus/diestrus frequency, and an increase in Fertile Stage index (FS), suggesting a prevention of the accelerated reproductive aging in TG female mice. However, a limitation of the current study is that faecal samples were collected exclusively during the metestrus and diestrus stages. Future research should examine the interactions among sex hormone levels, gut microbiota, and β-glucuronidase throughout all stages of the estrous cycle. Additionally, measuring sex hormone concentrations and their metabolites in faecal samples is necessary to determine the possible hormonal spectrum that a soluble fiber intervention may modulate. Furthermore, metatranscriptomics or validated functional strains analysis is essential to clarify the direct link between specific taxa, enzymatic activity, and host hormonal modulation.

Given the observed trend toward restored plasma estradiol levels and estrous cycle staging, we assessed estrobolome function by measuring β-glucuronidase activity in faecal samples. Notably, β-glucuronidase activity was higher in fructans-treated mice compared with their control groups (Fig. 3F), an effect accompanied by an increased abundance of β-glucuronidase-carrying bacteria in the faecal samples of F-treated mice (Fig. 5). Among the β-glucuronidase-carrying bacteria, Bacteroidota (Pollet et al. 2017) and *Bacteroides_H_857956* (Wang et al. 2021) showed the most significant increase in soluble fiber fed mice, tripling their relative abundance compared to their respective WT-C and TG-C control groups. In addition, *Lepagella*,* UBA3263* (both taxa belonging to the Bacteroidota phylum), *Akkermansia*, and *Faecalibaculum* were highly increased in soluble fiber-fed mice (Fig. 5B left side panel). A novel β-glucuronidase enzyme with a unique, highly active N-terminal loop has been described in *Akkermansia muciniphila* (Tarushi et al. 2025). *Feacalibacterium* is also a well-known β-glucuronidase-carrying bacterium (Pollet et al. 2017; Pellock et al. 2019). As previously reported, *Lactobacillus* and *Limosilactobacillus* were both reduced in TG-C compared to WT-C female mice (Romero-Flores et al. 2025). However, those two taxa showed a further reduction after soluble fiber intake, in both WT and TG female mice.

Spearman correlation analysis suggested that the abundance of specific taxa were associated with some physiological measurements evaluated in the animals, such as increased metestrus (*Turicibacter*); decreased estrus (*Cuitbacterium*), and proestrus frequencies (*UBA5905*,* Kineothrix*,* Fimenecus*,* Bifidobacterium_388775*); decreased DHEA (*Enterenecus*,* UBA3263*,* Avispirillum*) and progesterone levels (*Ligilactobacillus*,* Emergencia*,* Streptococcus*,* Limosilactobacillus*,* Lactococcus A_346120*); increased propionate (*Lactococcus A_346120)*, and acetate *(Turicibacter)* levels, further demonstrating that soluble fiber intake modulates the reproductive health in female mice.

A main feature of AD mouse models is the presence of Aβ plaques in the brain. It has been demonstrated that antibiotic treatment-induced changes in the gut microbiota reduced both the number and size of Aβ plaques in male APP/PS1 mice (Minter et al. 2016; Dodiya et al. 2019). We recently showed that one-month antibiotic treatment in six-month APP/PS1 female mice reduces Aβ plaques in posterior cortical regions (retrosplenial, auditory, and visual cortices), but not in the ventral hippocampus (Romero-Flores et al. 2025). In our current study, TG female mice fed with fructans did not exhibit significant changes in the number of Aβ plaques in the dorsal hippocampus or adjacent cortical areas (V2L and AuD); however, the size of Aβ plaques in the cortex was reduced in TG-F compared to TG-C female mice. Aβ aggregation in the colon has been observed in other AD mouse models (Puig et al. 2015). Here, we observed positive Aβ-IR in the colon of TG mice, with a significant reduction and restoration of the intestinal epithelial layer noted in TG-F females (Fig. 4A and B). Despite no overall change in Aβ plaque numbers in the brain, reduced plaque size in cortical areas of TG-F female mice could be associated with improvements in cognitive performance in F-treated mice. This data suggests that factors beyond Aβ plaque accumulation in the brain may play a critical role in enhancing cognitive function. For example, fructan intake reduced astrocyte activation in male APP/PS1 mice (Cuervo-Zanatta et al. 2023), and bioactive food interventions promote synaptic plasticity, attenuate neuroinflammation (Syeda et al. 2018), and maintain intestinal barrier integrity (Dalile et al. 2019; Ji et al. 2025). Given the significant cognitive improvement observed in TG-F female mice, additional evaluations of neuroinflammation, oxidative stress, and synaptic plasticity markers should be conducted to better define the underlying mechanisms of action of the dietary intervention. However, we measured faecal SCFAs levels as a direct measurement of gut bacterial fermentation. Faecal concentrations of butyrate and acetate were low in TG-C female mice, but these levels increased significantly after the intake of fructans. SCFAs profile differences between groups are particularly relevant given that butyrate exerts neuromodulatory effects and has been associated with neuroprotective and anti-inflammatory properties (Govindarajan et al. 2011; Guo et al. 2023). In contrast, the increased concentration of propionate in TG-C mice and the significant decrease following the consumption of dietary soluble fiber, are relevant, as propionate has been associated with metabolic dysfunction in astrocytes (Cuervo-Zanatta et al. 2023). *Faecalibaculum* and *Akkermansia* are well-known butyrate producers (O’Callaghan and van Sinderen 2016; Effendi et al. 2022), and were among the favoured bacterial *taxa* enriched in TG-F and WT-F female mice. *Lactobacillus*, a propionate producer, was reduced after the intake of dietary soluble fiber (Fig. 5B). Previously, we showed that the same dietary intervention (5% fructans in the diet) in male APP/PS1 rescued memory impairments and decreased neuroinflammation, both effects associated with changes in gut microbiota compositions. Specifically, there was a decreased relative abundance of *Lactobacillus spp* (propionate producer), and an increase in *Bacteroides* and several other taxa, well known for being butyrate producers (Cuervo-Zanatta et al. 2023). Another study showed that a 9-month bioactive food diet in 3xTGAD female mice significantly decreased *Lactobacillus ruminis* and increased *the relative abundances of Faecalibaculum and Akkermansia*. Those microbial changes were accompanied by increased butyrate concentration in faecal samples and decreased propionate levels in faecal and brain samples (Syeda et al. 2018). In addition, we recently showed that six-month-old female APP/PS1 mice also had low faecal butyrate concentrations (Romero-Flores et al. 2025). Thus, consuming 5% fructans for 2 months promotes the presence of specific gut bacteria, increasing butyrate and acetate production while simultaneously reducing propionate levels in TG female mice.

We used gut microbiota profiles to make functional predictions with PICRUSt. There were metabolic pathways differentially affected between groups, such as those involved in the production and maintenance of GABA supply (GABA shunt I) (Olsen and DeLorey 1999), which were higher in WT-C than in TG-C mice. Alterations in GABAergic signalling have been described in AD mice and AD patients (Lynex et al. 2004; Salazar et al. 2021). On the other hand, TG-F female mice show an increase in pathways related to Vitamin B6 biosynthesis, 2-oxobutanoate degradation I, phospholipases, pyridoxal 5’-phosphate (PLP) biosynthesis I, TCA cycle, L-methionine biosynthesis, menaquinol-9 and menaquinol-10 (Vitamin K2) biosynthesis, and 4-amino-2-methyl-5-diphosphomethylpyrimidin biosynthesis compared to TG-C female mice (Fig. 6B). PLP biosynthesis produces the active form of vitamin B6. Vitamin B6 is a vital micronutrient involved in one-carbon metabolism and is critical for remodelling the microbiota composition and its metabolic capacity (Hellmann and Mooney 2010; Mayengbam et al. 2020). The 4-amino-2-methyl-5-diphosphomethylpyrimidine pathway, which serves as a precursor for vitamin B1 (thiamine), was found to be elevated in TG-F mice. Vitamin B1 significantly influences the survival and competition of symbiotic bacteria. Additionally, vitamin B1 plays a role in the production of butyrate and acetate by the gut microbiota (Park et al. 2022). The TCA cycle was also enhanced in F-fed mice. Tea polyphenol intake also enhanced mitochondrial TCA cycle activity that depends on the gut microbiota and improved carbohydrate metabolism (Zhou et al. 2020). L-methionine, biosynthesised from homocysteine (Sobieszczuk-Nowicka et al. 2022), was increased after soluble fiber intake. High plasma levels of homocysteine are considered a risk factor for dementia and AD (Luchsinger et al. 2004). Thus, promoting the conversion to methionine may reduce homocysteine accumulation and dementia risk. Vitamin K2 has been associated with better cognitive function mainly due to its role in blood coagulation (Roumeliotis et al. 2025). The Vitamin K2 pathway was also increased in TG-F mice.

Our current findings indicate a potential connection between fructan consumption, estrobolome function, and cognitive and hormonal modulation in mice. We confirm previous findings that gut dysbiosis in female TG mice negatively impacts their circulating reproductive hormone levels, likely due to a compromised estrobolome. In addition, our current data strongly indicate that two months of fructan consumption led to significant interactions among gut bacteria. These interactions support the survival of specific bacterial taxa that express β-glucuronidase, produce SCFAs, and enhance multiple metabolic pathways related to reproductive health and cognitive function. Furthermore, the metabolic byproducts produced by gut microbiota during the breakdown of dietary soluble fiber may have complex metabolic and cellular effects at both systemic and central levels. However, the specific microbiota-derived mechanism associated with the brain and reproductive system remains to be determined.

Based on our findings, we strongly recommend increasing fructan intake as an effective strategy to significantly reduce the risk of Alzheimer’s disease in women.

### Limitations of the Study

This study provides new insights into the role of gut microbiota in the pathology of Alzheimer’s disease in females. It is important to note that this study did not explore the mechanisms underlying behavioural changes driven by the microbiota or their potential links to systemic effects and brain pathology associated with AD. This highlights the urgent need for further investigation.

## Supplementary Information

Below is the link to the electronic supplementary material.

Supplementary Material 1

## Data Availability

All sequencing data generated in this study are deposited at NCBI sequence read archive repository as bioproject [PRJNA718599] ([https://www.ncbi.nlm.nih.gov/Traces/study/?acc=PRJNA718599] (https:/www.ncbi.nlm.nih.gov/Traces/study/?acc=PRJNA718599) and https://www.ncbi.nlm.nih.gov/sra/?term=PRJNA718599), and are publicly available as of the date of publications. Any additional information required to reanalyze the data reported in this paper is available from the lead contact upon considerable request.
