# Supplementary material for: Soluble Fiber Intake Restores the Estrous Cycle and Sex Hormone Alterations in APP/PS1 Female Mice
Source: Cell Mol Neurobiol. 2026 May 20;46:131. doi: 10.1007/s10571-026-01745-3 (PMC13481976; doi:10.1007/s10571-026-01745-3)
Supplement: Supplementary file 1 [file 10571_2026_1745_MOESM1_ESM.docx]

**Table S1.** Relative abundance at phylum level in the experimental groups

| **Phylum** | **Group** | **%** | **SD** | **%CV** | **Range** |  | ***p*-value** |
| --- | --- | --- | --- | --- | --- | --- | --- |
| **Actinomycetota** | WT-C | 14.36 | 6.17 | 77.68 | 1.98 – 20.13 | **WT-C *vs.* WT-F** | 0.0049 ** |
|  | WT-F | 6.17 | 3.27 | 56.45 | 0.66 – 9.58 | TG-C *vs.* TG-F | 0.0938 |
|  | TG-C | 5.39 | 3.09 | 114.20 | 0.32 – 7.73 | **WT-C *vs.* TG-C** | < 0.0001 **** |
|  | TG-F | 5.34 | 3.31 | 66.30 | 0.69 – 8.28 | WT-F *vs.* TG-F | 0.9056 |
| **Bacteroidota** | WT-C | 1.96 | 0.92 | 54.39 | 0.22 – 2.76 | **WT-C *vs.* WT-F** | 0.0311 * |
|  | WT-F | 10.34 | 3.64 | 31.18 | 7.40 – 16.06 | **TG-C *vs.* TG-F** | 0.0014 ** |
|  | TG-C | 0.35 | 0.47 | 43.25 | 0.50 – 1.59 | WT-C *vs.* TG-C | 0.9242 |
|  | TG-F | 23.36 | 11.39 | 66.75 | 2.07 – 28.25 | WT-F *vs.* TG-F | 0.2949 |
| **Bacillota_A_368345** | WT-C | 24.55 | 9.63 | 47.98 | 4.75 – 34.43 | WT-C *vs.* WT-F | > 0.9999 |
|  | WT-F | 36.62 | 25.95 | 107.00 | 2.40 – 56.86 | TG-C *vs.* TG-F | 0.9743 |
|  | TG-C | 36.56 | 24.41 | 117.9 | 0.37 – 52.35 | WT-C *vs.* TG-C | 0.7226 |
|  | TG-F | 27.96 | 14.97 | 73.48 | 2.92 – 43.40 | WT-F *vs.* TG-F | 0.9166 |
| **Bacillota_I** | WT-C | 56.89 | 18.63 | 41.24 | 22.89 – 79.78 | **WT-C *vs.* WT-F** | 0.0003 *** |
|  | WT-F | 39.70 | 24.56 | 88.97 | 1.89 – 61.64 | TG-C *vs.* TG-F | 0.0898 |
|  | TG-C | 54.82 | 29.99 | 76.95 | 14.24 -78.50 | **WT-C *vs.* TG-C** | 0.0099 ** |
|  | TG-F | 36.54 | 20.52 | 88.48 | 0.50 – 56.73 | WT-F *vs.* TG-F | 0.7280 |
| **Verrucomicrobiota** | WT-C | 1.74 | 0.88 | 176.80 | 0 – 2.45 | WT-C *vs.* WT-F | 0.0971 |
|  | WT-F | 6.72 | 3.95 | 100.50 | 0.48 – 10.44 | **TG-C *vs.* TG-F** | 0.0334 * |
|  | TG-C | 2.66 | 1.60 | 74.82 | 0.04 – 3.81 | WT-C *vs.* TG-C | 0.9998 |
|  | TG-F | 6.12 | 3.87 | 87.89 | 0.74 – 9.50 | WT-F *vs.* TG-F | 0.9426 |
| **Other** | WT-C | 0.49 | 0.09 | 298.40 | 0 – 0.69 | WT-C *vs.* WT-F | 0.4073 |
|  | WT-F | 0.45 | 0.09 | 354.80 | 0 – 0.70 | TG-C *vs.* TG-F | 0.6472 |
|  | TG-C | 0.21 | 0.18 | 372.70 | 0 – 1.26 | WT-C *vs.* TG-C | 0.9522 |
|  | TG-F | 0.69 | 0.18 | 319.40 | 0 – 1.07 | **WT-F *vs.* TG-F** | 0.0073 ** |

Two-way ANOVA with Tukey’s post-hoc correction. Statistical significances are shown as **p*<0.05, ***p*<0.01, ****p*<0.001, *****p*<0.0001.

**Table S2.** Relative abundance at genus level in the experimental groups

| **Genus** | **Group** | **%** | **SD** | **%CV** | **Range** |  | ***p*-value** |
| --- | --- | --- | --- | --- | --- | --- | --- |
| ***Akkermansia*** | WT-C | 0.50 | 0.88 | 176.8 | 0 – 2.45 | **WT-C *vs.* WT-F** | 0.0195 * |
|  | WT-F | 4.60 | 3.37 | 73.34 | 2.24 – 10.44 | **TG-C *vs.* TG-F** | 0.0338 * |
|  | TG-C | 2.13 | 1.60 | 74.82 | 0.04 – 3.81 | WT-C *vs.* TG-C | 0.9998 |
|  | TG-F | 4.40 | 3.87 | 87.89 | 0.74 – 9.50 | WT-F *vs.* TG-F | 0.9952 |
| ***Bacteroides_H_857956*** | WT-C | 0 | 0 | 0 | 0 | WT-C *vs.* WT-F | 0.3667 |
|  | WT-F | 1.10 | 0.65 | 58.50 | 0.31 – 1.98 | **TG-C *vs.* TG-F** | 0.0035 ** |
|  | TG-C | 0 | 0 | 0 | 0 | WT-C *vs.* TG-C | 0.9907 |
|  | TG-F | 3.02 | 2.55 | 84.32 | 0.79 – 6.87 | WT-F *vs.* TG-F | 0.0625 |
| ***Bifidobacterium_388775*** | WT-C | 5.00 | 4.51 | 90.08 | 0 – 13.96 | **WT-C *vs.* WT-F** | < 0.0001 **** |
|  | WT-F | 1.33 | 1.51 | 113.50 | 0.07 – 3.45 | TG-C *vs.* TG-F | 0.9533 |
|  | TG-C | 1.97 | 2.57 | 130.30 | 0.02 – 5.83 | **WT-C *vs.* TG-C** | < 0.0001 **** |
|  | TG-F | 1.92 | 2.76 | 143.60 | 0.03 – 6.68 | WT-F *vs.* TG-F | 0.8339 |
| ***Dubosiella*** | WT-C | 1.64 | 4.60 | 280.80 | 0 – 13.01 | WT-C *vs.* WT-F | 0.5312 |
|  | WT-F | 0 | 0 | 0 | 0 | TG-C *vs.* TG-F | 0.9998 |
|  | TG-C | 0 | 0 | 0 | 0 | WT-C *vs.* TG-C | 0.5296 |
|  | TG-F | 0 | 0 | 0 | 0 | WT-F *vs.* TG-F | > 0.9999 |
| ***Duncaniella*** | WT-C | 0 | 0 | 0 | 0 | WT-C *vs.* WT-F | > 0.9999 |
|  | WT-F | 0 | 0 | 0 | 0 | **TG-C *vs.* TG-F** | 0.0022 ** |
|  | TG-C | 2.00 | 1.54 | 76.95 | 0.72 – 4.59 | **WT-C *vs.* TG-C** | 0.0022 ** |
|  | TG-F | 0 | 0 | 0 | 0 | WT-F *vs.* TG-F | > 0.9999 |
| ***Faecalibaculum*** | WT-C | 0 | 0 | 0 | 0 | **WT-C *vs.* WT-F** | < 0.0001 **** |
|  | WT-F | 19.44 | 4.91 | 25.23 | 10.53 – 24.63 | TG-C *vs.* TG-F | 0.3927 |
|  | TG-C | 0 | 0 | 0 | 0 | WT-C *vs.* TG-C | 0.9680 |
|  | TG-F | 1.88 | 3.21 | 170.6 | 0 – 8.29 | **WT-F *vs.* TG-F** | < 0.0001 **** |
| ***Fimenecus*** | WT-C | 5.87 | 4.41 | 75.23 | 1.30 – 12.67 | **WT-C *vs.* WT-F** | < 0.0001 **** |
|  | WT-F | 0.66 | 0.39 | 58.98 | 0.01 -1.04 | TG-C *vs.* TG-F | 0.9852 |
|  | TG-C | 2.42 | 2.64 | 108.90 | 0.13 – 6.31 | **WT-C *vs.* TG-C** | < 0.0001 **** |
|  | TG-F | 2.16 | 2.58 | 119.50 | 0 – 6.20 | WT-F *vs.* TG-F | 0.8955 |
| ***Ileibacterium*** | WT-C | 0 | 0 | 0 | 0 | WT-C *vs.* WT-F | 0.2263 |
|  | WT-F | 8.16 | 8.85 | 108.50 | 0.92 – 22.54 | TG-C *vs.* TG-F | 0.3016 |
|  | TG-C | 12.58 | 16.74 | 133.10 | 0 – 38.68 | WT-C *vs.* TG-C | 0.0662 |
|  | TG-F | 0.70 | 0.70 | 202.00 | 0 – 3.24 | WT-F *vs.* TG-F | 0.7040 |

**(...continuation) Table S2.** Relative abundance at genus level in the experimental groups

| **Genus** | **Group** | **%** | **SD** | **%CV** | **Range** |  | ***p*-value** |
| --- | --- | --- | --- | --- | --- | --- | --- |
| ***Lactobacillus*** | WT-C | 25.79 | 16.03 | 62.10 | 4.13 – 55.17 | **WT-C *vs.* WT-F** | < 0.0001 **** |
|  | WT-F | 7.37 | 9.12 | 123.70 | 0.18 – 23.63 | TG-C *vs.* TG-F | 0.5043 |
|  | TG-C | 17.18 | 18.56 | 108.00 | 0.28 – 47.05 | **WT-C *vs.* TG-C** | 0.0003 *** |
|  | TG-F | 5.88 | 5.13 | 87.22 | 0.10 – 14.08 | WT-F *vs.* TG-F | 0.6352 |
| ***Lactococcus_A_346120*** | WT-C | 3.63 | 2.14 | 58.89 | 0.85 – 7.94 | **WT-C *vs.* WT-F** | 0.0001 *** |
|  | WT-F | 1.56 | 1.49 | 95.67 | 0.35 – 3.91 | TG-C *vs.* TG-F | 0.1546 |
|  | TG-C | 3.42 | 2.39 | 70.00 | 0.23 – 5.95 | WT-C *vs.* TG-C | 0.0641 |
|  | TG-F | 1.39 | 0.89 | 64.15 | 0.16 – 2.21 | WT-F *vs.* TG-F | 0.8174 |
| ***Lawsonibacter*** | WT-C | 2.70 | 1.88 | 69.83 | 0 – 5.61 | WT-C *vs.* WT-F | 0.3086 |
|  | WT-F | 2.49 | 3.24 | 130.10 | 0.09 – 7.53 | TG-C *vs.* TG-F | 0.5463 |
|  | TG-C | 1.83 | 1.93 | 105.50 | 0.05 – 4.71 | **WT-C *vs.* TG-C** | 0.0120 * |
|  | TG-F | 2.16 | 1.74 | 80.24 | 0.35 – 5.16 | WT-F *vs.* TG-F | 0.9131 |
| ***Lepagella*** | WT-C | 0 | 0 | 0 | 0 | WT-C *vs.* WT-F | 0.1203 |
|  | WT-F | 7.86 | 3.92 | 49.88 | 4.30 – 12.78 | **TG-C *vs.* TG-F** | 0.0187 * |
|  | TG-C | 0.01 | 0.02 | 200.00 | 0 – 0.04 | WT-C *vs.* TG-C | 0.9997 |
|  | TG-F | 13.32 | 11.81 | 88.66 | 0.25 – 28.91 | WT-F *vs.* TG-F | 0.3670 |
| ***Ligilactobacillus*** | WT-C | 1.86 | 0.69 | 36.80 | 0.63 – 2.56 | **WT-C *vs.* WT-F** | < 0.0001 **** |
|  | WT-F | 0 | 0 | 0 | 0 | **TG-C *vs.* TG-F** | < 0.0001 **** |
|  | TG-C | 1.30 | 0.52 | 39.62 | 0.67 – 1.94 | **WT-C *vs.* TG-C** | 0.0003 *** |
|  | TG-F | 0 | 0 | 0 | 0 | WT-F *vs.* TG-F | > 0.9999 |
| ***Limosilactobacillus*** | WT-C | 4.11 | 2.50 | 60.48 | 0.96 – 8.20 | **WT-C *vs.* WT-F** | < 0.0001 **** |
|  | WT-F | 0 | 0 | 0 | 0 | **TG-C *vs.* TG-F** | 0.0183 * |
|  | TG-C | 2.77 | 1.97 | 71.08 | 0.05 – 4.50 | **WT-C *vs.* TG-C** | 0.0324 * |
|  | TG-F | 0 | 0 | 0 | 0 | WT-F *vs.* TG-F | > 0.9999 |
| ***Roseburia_A_166204*** | WT-C | 0 | 0 | 0 | 0 | WT-C *vs.* WT-F | 0.5166 |
|  | WT-F | 1.71 | 4.08 | 238.70 | 0 – 10.04 | TG-C *vs.* TG-F | 0.9997 |
|  | TG-C | 0 | 0 | 0 | 0 | WT-C *vs.* TG-C | 0.9997 |
|  | TG-F | 0 | 0 | 0 | 0 | WT-F *vs.* TG-F | 0.5166 |
| ***Turicibacter*** | WT-C | 0.62 | 0.74 | 120.30 | 0 – 1.75 | **WT-C *vs.* WT-F** | 0.0112 * |
|  | WT-F | 0 | 0 | 0 | 0 | TG-C *vs.* TG-F | 0.9960 |
|  | TG-C | 0 | 0 | 0 | 0 | **WT-C *vs.* TG-C** | 0.0112 * |
|  | TG-F | 0 | 0 | 0 | 0 | WT-F *vs.* TG-F | > 0.9999 |

**(...continuation) Table S2.** Relative abundance at genus level in the experimental groups

| **Genus** | **Group** | **%** | **SD** | **%CV** | **Range** |  | ***p*-value** |
| --- | --- | --- | --- | --- | --- | --- | --- |
| ***UBA3263*** | WT-C | 0 | 0 | 0 | 0 | WT-C *vs.* WT-F | > 0.9999 |
|  | WT-F | 0 | 0 | 0 | 0 | **TG-C *vs.* TG-F** | 0.0031 ** |
|  | TG-C | 0 | 0 | 0 | 0 | WT-C *vs.* TG-C | 0.9978 |
|  | TG-F | 1.03 | 0.78 | 75.60 | 0.37 – 2.47 | **WT-F *vs.* TG-F** | 0.0026 ** |
| ***UBA3282*** | WT-C | 1.71 | 3.43 | 200.10 | 0 – 10.16 | WT-C *vs.* WT-F | 0.9132 |
|  | WT-F | 3.45 | 5.69 | 165.00 | 0 – 14.72 | TG-C *vs.* TG-F | 0.9702 |
|  | TG-C | 5.11 | 10.74 | 210.10 | 0.03 – 24.30 | WT-C *vs.* TG-C | 0.6964 |
|  | TG-F | 5.28 | 7.53 | 142.70 | 0.14 – 18.27 | WT-F *vs.* TG-F | 0.7588 |
| **Other** | WT-C | 0.09 | 0.34 | 363.50 | 0 – 5.41 | **WT-C *vs.* WT-F** | < 0.0001 **** |
|  | WT-F | 0.09 | 0.31 | 341.90 | 0 – 2.99 | **TG-C *vs.* TG-F** | < 0.0001 **** |
|  | TG-C | 0.06 | 0.28 | 442.00 | 0 – 4.53 | **WT-C *vs.* TG-C** | < 0.0001 **** |
|  | TG-F | 0.09 | 0.32 | 359.70 | 0 – 3.06 | WT-F *vs.* TG-F | 0.9380 |

Two-way ANOVA with Tukey’s post-hoc correction. Statistical significances are shown as **p*<0.05, ***p*<0.01, ****p*<0.001, *****p*<0.0001.

**Table S3.** Alpha diversity indixes in the experimental groups.

| **Indixes** | **Group** | **%** | **SD** | **%CV** | **Range** | **Group** | ***p*-value** |
| --- | --- | --- | --- | --- | --- | --- | --- |
| **CHAO1** | WT-C | 70.43 | 14.23 | 20.21 | 44.0 – 90.0 | WT-C *vs.* WT-F | 0.2371 |
|  | WT-F | 89.40 | 10.50 | 11.75 | 75.0 – 102.0 | TG-C *vs.* TG-F | > 0.9999 |
|  | TG-C | 75.20 | 12.87 | 17.12 | 60.0 – 94.0 | WT-C *vs.* TG-C | 0.9326 |
|  | TG-F | 75.60 | 10.16 | 13.44 | 62.0 – 85.0 | WT-F *vs.* TG-F | 0.5471 |
| **ACE** | WT-C | 70.43 | 14.23 | 20.21 | 44.0 – 90.0 | WT-C *vs.* WT-F | 0.2371 |
|  | WT-F | 89.40 | 10.50 | 11.75 | 75.0 – 102.0 | TG-C *vs.* TG-F | > 0.9999 |
|  | TG-C | 75.20 | 12.87 | 17.12 | 60.0 – 94.0 | WT-C *vs.* TG-C | 0.9326 |
|  | TG-F | 75.60 | 10.16 | 13.44 | 62.0 – 85.0 | WT-F *vs.* TG-F | 0.5471 |
| **Shannon** | WT-C | 2.16 | 0.66 | 30.50 | 1.27 – 2.84 | **WT-C *vs.* WT-F** | 0.0499 * |
|  | WT-F | 3.13 | 0.52 | 16.52 | 2.58 – 3.72 | **TG-C *vs.* TG-F** | 0.0041 ** |
|  | TG-C | 1.70 | 0.61 | 35.66 | 1.02 – 2.38 | WT-C *vs.* TG-C | 0.4449 |
|  | TG-F | 3.05 | 0.36 | 11.94 | 2.62 – 3.59 | WT-F *vs.* TG-F | 0.9946 |
| **Simpson** | WT-C | 0.83 | 0.09 | 10.62 | 0.67 – 0.94 | **WT-C *vs.* WT-F** | 0.0191 * |
|  | WT-F | 0.95 | 0.04 | 4.22 | 0.87 – 0.97 | TG-C *vs.* TG-F | 0.5285 |
|  | TG-C | 0.85 | 0.07 | 8.45 | 0.74 – 0.91 | WT-C *vs.* TG-C | 0.8351 |
|  | TG-F | 0.90 | 0.05 | 5.64 | 0.81 – 0.95 | WT-F *vs.* TG-F | 0.6580 |
| **Fisher** | WT-C | 9.15 | 2.35 | 25.66 | 5.42 – 11.27 | **WT-C *vs.* WT-F** | 0.0181 * |
|  | WT-F | 14.64 | 2.01 | 13.71 | 11.05 – 16.69 | TG-C *vs.* TG-F | 0.9989 |
|  | TG-C | 10.77 | 3.30 | 30.60 | 7.23 – 16.74 | WT-C *vs.* TG-C | 0.8163 |
|  | TG-F | 10.99 | 2.77 | 25.15 | 8.33 – 16.10 | WT-F *vs.* TG-F | 0.1223 |

Two-way ANOVA with Tukey’s post-hoc correction. Statistical significances are shown as **p*<0.05, ***p*<0.01, ****p*<0.001, *****p*<0.0001.

**Supplementary Figure S1.**

12. Superpathway of D-glucarate and

D-galactarate degradation

13. L-histidine degradation III

14. L-histidine degradation I

12.

13.

14.

**WT-C vs. WT-F**

**7.** Formaldehyde assimilation II (assimilatory RuMP Cycle)

**8.** Superpathway of glycerol degradation to 1,3-propanediol

**9.** CO2 fixation

**10.** L-arginine degradation XIV

**11.** L-histidine degradation II

**WT-F *vs*. TG-F**

**10.**

**11.**

**7.**

**8.**

**9.**

**Figure S1. Phylogenetic Investigation of Communities by Reconstruction of Unobserved States (PICRUST) analysis.** Bar plots showing MetaCyc pathways classification of PICRUST that were significantly different (P < 0.05) between:  WT-C vs. WT-F and WT-F vs. TG-F. WT-C n = 8, WT-F n = 6, and TG-F n = 6. Comparisons were performed using Welch t-test applied False Discovery Rate (FDR) correction.

**Supplementary Figure S2.**

**A)** **Elevated Plus maze test** **B)** **T-Maze test**

**Suppl. Fig S2**. Locomotor activity was not directly assessed in the animals. However, total exploration time (sec) and the percentage of time spent in the center (%) of the Elevated Plus Maze (EPM) **(A)**, and the total arm entries in the T-maze test **(B)** were evaluated. There were no differences among the groups in any of the evaluations. For A) WT-C n = 7, WT-F n = 6, TG-C n = 6, and TG-F n = 6. For B) WT-C n = 7, WT-F n = 7, TG-C n = 5, and TG-F n = 7. The data are shown as mean ± standard deviation (SD). Two-way ANOVA with Tukey’s post hoc correction was used.

**Supplementary Figure S3.**

Western blots for Estrogen Receptor alpha (ERα) obtained from cortical samples
